# Two-dimensional TIRF-SIM–traction force microscopy (2D TIRF-SIM-TFM)

**DOI:** 10.1038/s41467-021-22377-9

**Published:** 2021-04-12

**Authors:** Liliana Barbieri, Huw Colin-York, Kseniya Korobchevskaya, Di Li, Deanna L. Wolfson, Narain Karedla, Falk Schneider, Balpreet S. Ahluwalia, Tore Seternes, Roy A. Dalmo, Michael L. Dustin, Dong Li, Marco Fritzsche

**Affiliations:** 1 MRC Human Immunology Unit, Weatherall Institute of Molecular Medicine, University of Oxford, Oxford, UK; 2 Kennedy Institute for Rheumatology, University of Oxford, Oxford, UK; 3 National Laboratory of Biomacromolecules, Institute of Biophysics, Chinese Academy of Sciences, Beijing, China; 4 Department of Physics and Technology, UiT The Arctic University of Norway, Tromsø, Norway; 5 Rosalind Franklin Institute, Didcot, UK; 6 Norwegian College of Fishery Science, UiT The Arctic University of Norway, Tromsø, Norway; 7 College of Life Sciences, University of Chinese Academy of Sciences, Beijing, China

**Keywords:** Biophysical methods, Imaging, Fluorescence imaging, Biophysics

## Abstract

Quantifying small, rapidly evolving forces generated by cells is a major challenge for the understanding of biomechanics and mechanobiology in health and disease. Traction force microscopy remains one of the most broadly applied force probing technologies but typically restricts itself to slow events over seconds and micron-scale displacements. Here, we improve >2-fold spatially and >10-fold temporally the resolution of planar cellular force probing compared to its related conventional modalities by combining fast two-dimensional total internal reflection fluorescence super-resolution structured illumination microscopy and traction force microscopy. This live-cell 2D TIRF-SIM-TFM methodology offers a combination of spatio-temporal resolution enhancement relevant to forces on the nano- and sub-second scales, opening up new aspects of mechanobiology to analysis.

## Abstract (web summary)

Quantifying rapid and small cellular forces is a major challenge in mechanobiology. Here, the authors show a >2-fold spatially and >10-fold temporally force sampling improvement combining traction force microscopy with total internal reflection fluorescence super-resolution structured illumination microscopy.

## Introduction

Mechanical forces guide the function of living cells^1,2^. Although the biological effects of biomechanics have perhaps always been most evident in the context of the physical behaviour of cells, it has become apparent that mechanical forces can direct the function of cells in many biological contexts in health and disease. New insights in the field of mechanobiology have revealed that cellular physiology is directly influenced by the mechanical properties and forces generated within the cellular environment^3–5^. The ability of cells to generate and respond to these mechanical cues are primarily processed by the actin cytoskeleton, a key determinant of cellular biomechanics, that comprises dynamic actin architectures undergoing continuous re-arrangements and turnover^3,6,7^. The strength of the forces generated by cells evolve in time, as does a cell’s sensitivity to changes in the magnitude, frequency and duration of the surrounding biomechanical stimuli^4^. Cells are thus able to adjust their biomechanics in order to meet their physiological needs^5,8,9^. Quantifying mechanical forces within the context of fast nanoscale dynamics of cortical actin architectures has therefore become an important step in understanding the mechanobiology of living cells.

Traction force microscopy (TFM) has become one of the most commonly applied force probing methodologies^10–12^, primarily due to its adaptability in modelling biological and mechanical properties, ease of implementation, and reliance on widely available materials and fluorescence microscopy^13^. Despite its importance and proven biological significance, quantifying the dynamic process of force production in living cells via TFM remains challenging due to technical constraints. Efforts aiming to approach physiological sensitivity have led to combinations of TFM and different optical microscopy modalities with extended spatial and temporal resolution^11,12^. Nevertheless, the sensitivity and biocompatibility of TFM have remained dependent on the individually chosen imaging modality, highlighting the difficulty of overcoming the trade-off between spatial resolution, temporal resolution, and acquisition duration^14–16^. Despite an urgent demand from the biological context, these limitations have thus far precluded TFM in its application to biological questions involving cellular force production exhibiting both sub-micron length-scales and sub-second time-scales^17^. For example, in the context of immune cell activation, mechanical forces generated by immune cells have been implicated in their function, playing a role in antigen recognition and discrimination^18–20^. In addition, rapid force generation is vital for cell migration, for example, in the case of wound-healing processes^21–23^. Consequently, there is a pressing need to improve the sensitivity and biocompatibility of TFM to quantify these biomechanical forces of living cells.

In a TFM experiment, living cells are plated onto the topmost layer of a homogeneous, isotropic, elastic, thin (20–30 µm) gel substrate, deposited on a glass coverslip^13^. The molecular properties of the gel surface and its bulk mechanical properties can be adjusted to provide physiological surface conditions by tuning the surface protein functionalisation and gels stiffness, respectively. Both hydrogels and elastomers, such as polyacrylamide and polydimethylsiloxane (PDMS), respectively, are used in TFM experiments as elastic substrates with tunable stiffness and, in the case of PDMS, tunable refractive index allowing total internal reflection fluorescence (TIRF) microscopy on top of the substrate^24–26^. To extract the mechanical force of the cell, the gel deformation is tracked with respect to the relaxed state^24^. To accurately track the gel deformation, both reference and reference-free TFM methods are available, with the later made possible via a number of strategies, including the deposition of a structured pattern of quantum dot inks on the gel surface or two-photon laser scanning lithography^27–30^. Despite the advantage of not having to record a reference frame of the gels relaxed state, the reference-free methods require specialist equipment and are also limited in their lateral spatial resolution. A versatile reference-based TFM method that allows increased spatial resolution consists of coating the topmost gel layer with randomly distributed sub-diffraction-sized fluorescent marker beads. During cell–gel interactions, mechanical forces generated by the cell displaces the gel, and therefore the fluorescent marker beads. The beads displacement is monitored by optical fluorescence microscopy. Using a theoretical description of the gel substrate combined with knowledge of the elastic gel characteristics, both the magnitude and direction of the cellular mechanical forces can be calculated from the spatial displacements^10^.

The sensitivity of TFM is primarily determined by the spatial and temporal resolution of the microscopy modality. The frequency of spatial and temporal acquisition sampling must both be sufficiently high to enable probing at the length- and time-scale demanded by the biology. The finite diffraction limit of a fluorescence microscope imposes a fundamental upper limit on the bead densities in conventional fluorescence microscopy, profoundly constraining the spatial resolution of TFM. To partially address this constraint, Sabass et al.^10^ proposed to combine two different fluorescent beads to increase the bead density (see Table 1). We have recently established the 2D STED-TFM methodology by combining super-resolution stimulated emission depletion (STED) microscopy and TFM^31^. STED-TFM offers up to fivefold improvement in spatial sampling, extending the sensitivity of TFM. Nonetheless, because STED relies on beam scanning and temporal averaging at each pixel, the total acquisition speed is limited, practically restricting STED-TFM to 2D TFM with one to multiple seconds (s) per frame (see Table 1). In addition, STED requires high laser intensity for fluorescence depletion, minimising biocompatibility. Importantly, the slow acquisition duration and the low spatial resolution of cellular imaging also precludes the subsequent imaging and correlation of the dynamics of the cell architectures responsible for the mechanical force production. Stubb et al.^32^ addressed the enhancing of the spatial resolution of TFM applying fluorescence fluctuation analysis on fluorescent bead images acquired using both spinning disk confocal and widefield microscopy. Thanks to fluctuation-based analysis, they achieved a lateral spatial resolution of 200 nm but at the expense of a loss in temporal resolution to multiple seconds per frame (see Table 1). Extending these efforts, we next established the live-cell super-resolution 3D SIM-TFM methodology by combining structured illumination microscopy (SIM) and TFM^17^. Because SIM is a wide-field (WF) technique^33,34^, it does not rely on image raster scanning, but provides practically simultaneous super-resolved cell and force field acquisition, extending super-resolved 2D TFM to 3D TFM at comparable spatial and temporal sampling (80–100 nm lateral and 300 nm axial, see Table 1)^17^. More recently, by combing TFM with astigmatic imaging, aTFM enables rapid and highly sensitive estimation of both lateral and axial forces^35^. Note, for reasons of consistency all length-scale units are reported in nanometre (nm) values within the text but in both pixel (px) and nm units in the primary figures and supplementary figures.

**Table 1:** Summary of TFM methods based on fluorescent marker beads protocol compared to prior state-of-the-art methodologies.

	Lateral imaging spatial resolution	Axial imaging spatial resolution	Temporal resolution (frames per second)	Illumination power (kW/cm^2^)	TFM sensitivity (bead/µm^2^)	2D force estimation	3D force estimation
2D TIRF-SIM-TFM	<100 nm	n/a	1–10 fps^a^	0.02–0.1	15–20	✓	✗
aTFM^35^	200–300 nm	20 nm^b^	1–10 fps^a^ (2 µm *z* range)	0.02–0.1	0.3	✓	✓
Prior state of the art							
3D SIM-TFM^17^	<100 nm	250 nm	0.1–1 fps (2 µm *z* range)	0.02–0.1	1	✓	✓
2D STED-TFM^31^	<100 nm	300 nm	0.05–0.1 fps^c^	1–10^5^	2.2–5	✓	✗
TIRF	300	n/a	1 fps	0.02–0.1	1	✓	✗
FBSR^32^	200 nm	n/a	0.05 fps	Not reported	1.2	✓	✗
High-resolution TFM^10^	200–300 nm	600–800 nm	0.2 fps^c^	Not reported	Not reported	✓	✗
Confocal	200–300 nm	600–800 nm	0.1–1 fps	0.1–10	1	✓	✓

^a^The primary speed limitation of TIRF, 2D TIRF-SIM-TFM and aTFM is imposed by the camera exposure time. ^b^The value represents the axial localisation uncertainty in aTFM. ^c^The value indicates the frame rate for a FOV of about 10 µm^2^.

Moreover, in addition to the microscopy imaging modality, the sensitivity of TFM depends on the analysis strategy employed to extract the displacement information from the experimentally acquired fluorescent bead images^24^. Extraction of the bead displacements is typically achieved through single particle tracking (SPT) or techniques based on statistical comparisons of fluorescent images, such as particle image velocimetry (PIV)^24^. In both cases, extending the spatial resolution of the image acquisition modality results in an improved recovery of the displacement field and hence a more accurate determination of the stress field^13,31^. The SPT approach relies on localising each bead individually followed by the linking of individual localisations into trajectories. The quality of the tracking thereby depends on the localisation of each bead in space and time, thus for any given bead the displacement information can be resolved with high confidence. To achieve this, the SPT approach depends on the spatial resolution of the applied microscopy modality because spatially under-sampled distributions of fluorescent beads lead to overlapping beads and thus erroneous localisation. In contrast, the PIV approach does not limit itself to the trackability of fluorescent beads. The fluorescent image is divided into sub-regions, with so-called PIV windows, and compared between time-points using spatial correlation to assess the relative spatial shift of their fluorescence content. The quality of PIV depends on the size of the chosen PIV window, and care must be taken that a sufficient number of beads is present within each of those windows. Importantly, PIV is robust against overlapping beads, allowing a greater flexibility in the choice of experimental bead density^13^. In addition to the spatial resolution, the quality of SPT and PIV depends on the temporal resolution, where temporal under-sampling constrains the trackability of individual beads and reliable correlation of PIV windows. Consequently, a combination of optimal PIV window size and spatiotemporal resolution holds the potential to augment the best possible sensitivity for TFM.

Mechanical forces are present in many biological phenomena, and often evolve rapidly within seconds from small nanoscale to micron-scale forces as, for example, the dynamics of ventral 2D cell-surface interfaces during cancer cell adhesion, migration, and during immune cell activation^17,29,31,32,36–38^. For these reasons, there is a continuing demand to develop a sensitive TFM force probing modality for quantifying small, rapidly evolving, planar forces in cells on the sub-second time-scale and sub-micron spatial scale. Live-cell super-resolution total internal reflection fluorescence SIM (TIRF-SIM) has recently been highlighted to provide both extended spatial and temporal resolution at minimal invasiveness to the biology^7,33^. Historically, because the mechanical forces are detected at the top surface layer of the gel, image acquisition has previously required a microscope modality that can operate away from the coverslip, excluding TIRF as a suitable imaging modality^31^. However, as is required for TFM, the TIRF excitation can be moved to the upper gel layer by matching the refractive index of the gel substrate and the glass coverslip below, allowing TIRF-SIM illumination at the gel to media interface^25^. Notably, there is currently no other competitive fluorescence imaging modality available comparable to TIRF-SIM in terms of extended temporal and spatial sampling. Although for instance lattice light sheet microscopy provides fast temporal sampling^39^, it remains a volumetric technique which is diffraction-limited and slower than TIRF-SIM due to requirement of multi-frame z-stack acquisition.

Here, to enable sensitive physiological 2D force probing in living cells, we combine TFM with fast super-resolution high numerical aperture (NA) TIRF-SIM. Applying a combination of PIV-based computer simulations and live-cell experiments, we demonstrate the experimental power and sensitivity at >2-fold laterally increased spatial resolution and >10-fold enhanced temporal resolution as offered by TIRF-SIM compared to conventional TFM. This live-cell 2D TIRF-SIM-TFM methodology revealed small, rapidly evolving shear mechanical forces generated during cell–substrate adherence of cervical cancer cells, the early stages of activating immune rat basophilic leukaemia (RBL) cells, and the migration of primary salmon keratocytes (skin epithelial cells) with unprecedented spatiotemporal resolution, opening up new aspects of mechanobiology to analysis.

## Results

**Fig. 1:**
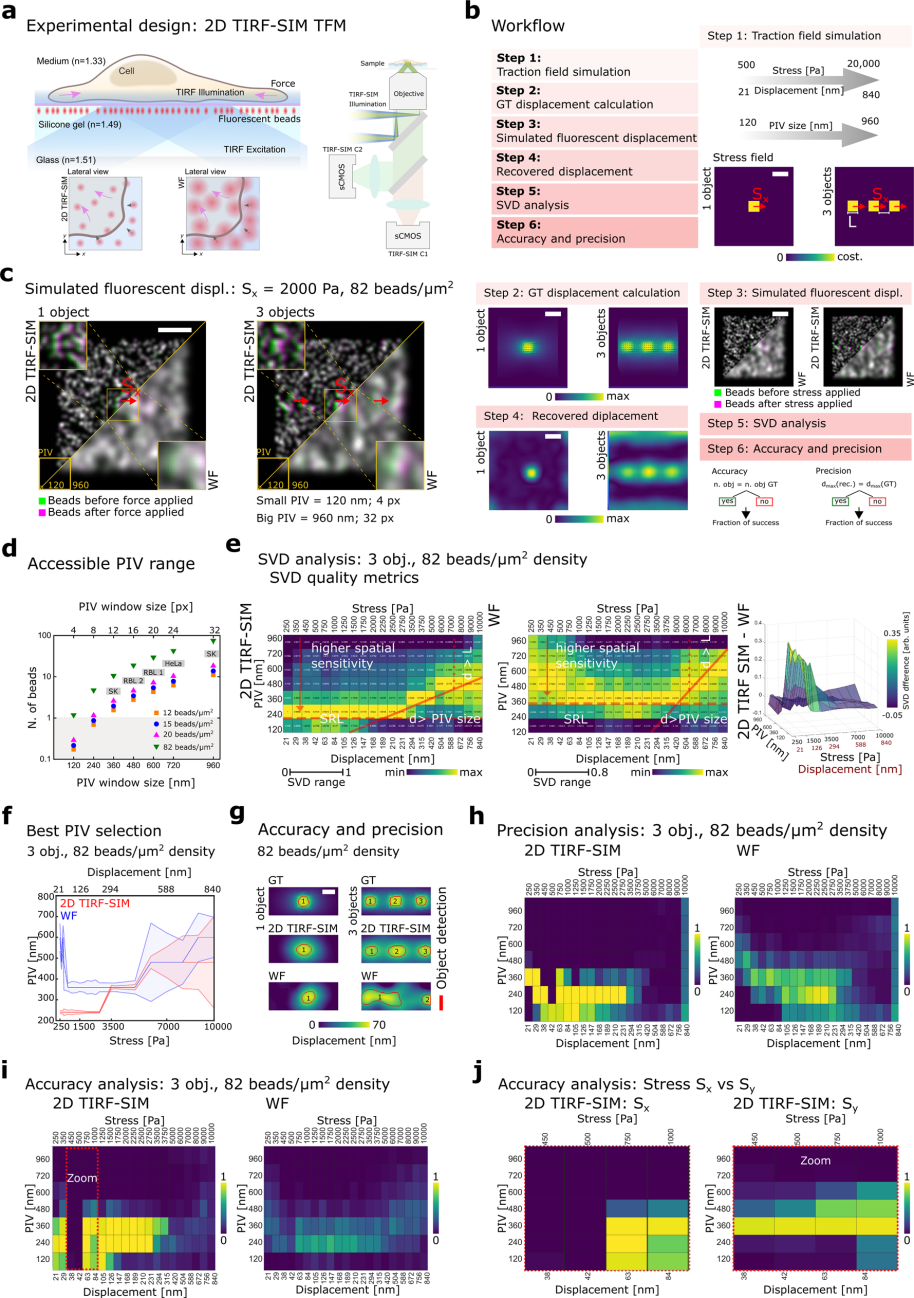
Overview of computer simulations as a guide for optimal TFM analysis. **a** Experimental design of the 2D TIRF-SIM-TFM. Left: TIRF excitation light at the interface between the gel and the cell. Right: Optical path of the TIRF-SIM platform. **b** Graphical diagram outlining the simulation pipeline. Scale bar: 1 µm. **c** Example of simulated fluorescent displacement image pairs for both the single- and triple square-shaped stress zones. Density of 82 beads/µm^2^. The images before (green) and after (magenta) a 1000 Pa lateral stress in the *x* direction are superimposed. Images are shown in 2D TIRF-SIM and WF with a zoom-in of the central part of the image. Scale bar: 1 µm. **d** Plot outlining the relationship between the PIV windows size and the expected number of beads within a given PIV window at different beads densities. SK, salmon keratocytes. **e** Left and centre- heat maps showing the quality of the displacement estimation compared to the GT for a range of PIV windows size and displacement magnitudes using the SVD quality metric at a bead density of 82 beads/μm^2^. Value of 1 representing the best quality. Each column is colour-scaled between minimum and maximum SVD values. SRL spatial resolution limit. Right: SVD quality metric difference between TIRF-SIM and WF. Plots normalised to the TIRF-SIM maximum. Values close to 1 indicating improved displacement estimation. **f** Plot guiding the choice of the optimal PIV windows size for a given displacement, based on the minimum SVD quality metric. Density of 82 beads/μm^2^. **g** Example of the binarisation of displacement fields for the accuracy and precision analysis (red line). Single and three squares cases are shown for both GT, 2D TIRF-SIM and WF modalities. Stress applied = 1000 Pa, PIV window = 360 nm. Density of 82 beads/µm^2^. Scale bar: 1 µm. **h** Precision maps for the three squares case. Density of 82 beads/µm^2^. The values show the fraction of success among 100 repeats. **i** Accuracy maps for the three squares case with 82 beads/µm^2^ density. **j** Comparison of accuracy maps for 2D TIRF-SIM case, for horizontal and vertical stress in the range between 450 and 1000 Pa. Density of 82 beads/µm^2^.

The 2D TIRF-SIM-TFM methodology combines TFM and an existing custom-built TIRF-SIM platform^17,25^. By applying the appropriate critical angle and matching the refractive index of the glass and gel interface, the TIRF field was moved to the topmost layer of the high refractive index (*n* = 1.49) silicone gel sample allowing simultaneous super-resolved acquisition of both the fluorescent cell and bead channel over time (Fig. 1a). Advantages of TIRF-SIM over 2D SIM image acquisition are all inherent to the isotropic nature of the TIRF point spread function (PSF; <100 ± 20 nm laterally and axially) in contrast to the non-isotropic PSF of 2D SIM (<100 ± 20 nm laterally and <300 ± 60 nm axially) maximising contrast in the fluorescent cell channel and minimising background and noise contributions. To take advantage of the provided increased spatiotemporal resolution of SIM, we chose the PIV analysis approach over SPT to maximise the bead density and thus the experimental power of the 2D TIRF-SIM-TFM technique as detailed in the following in silico analysis and live-cell experiments.

### Computer simulations as a guide for optimal TFM analysis

We started by establishing of an understanding of how the sensitivity of 2D TIRF-SIM-TFM depends on the spatiotemporal resolution of the TIRF-SIM image acquisition, the density of the fiducial marker beads, and the PIV analysis window size. Hence, we conducted computer simulations to determine the dynamic range of the sensitivity improvement of TFM comparing TIRF-SIM and WF image acquisition. For this, we first determined the optimal analysis parameters for use with PIV for a given set of in silico conditions. With the intent of quantifying these conditions, we built a simulation pipeline comprising four steps, including the simulation of the stress field, the calculation of the corresponding ground truth (GT) displacement field, the calculation of the respective simulated fluorescent image pairs as observed by TIRF-SIM and WF, and lastly, the determination of the recovered displacement field for a given PIV window size (Fig. 1b). The final recovered displacement fields were then quantified and interpreted by comparing to the GT via two independent metrics outlined in two additional analysis steps. The computer simulations were designed to mimic 2D TFM measurements in the presence of a well-defined force in the units of stress ranging from 250 Pa to 10 kPa using a 10 kPa reference gel sample with both a sparse and dense distribution of fiducial marker beads (see “Methods”). In the initial simulation step, a stress field of a well-defined shape was applied to a square domain of size 5 × 5 µm^2^ at a given elastic material stiffness of 10 kPa (Step 1, Fig. 1b). This computational domain size was chosen to minimise both the computational cost for displacement recovery and avoiding boundary effects due to the finite size of the stress field. Considering effects of both signal-to-noise and spatial resolution of TIRF-SIM and WF image acquisition, we compared simulations with a uniform distribution of stress in the *x* direction (*S*_*x*_) containing a single square-shaped stress zone of fixed size 0.2 × 0.2 µm^2^ or three collinear square-shaped stress zones of fixed size 0.2 × 0.2 µm^2^ equally separated by a distance *L* = 0.2 µm along the *x*-axis. Note, both terms “traction” and “stress” refer to the 2D force exerted by the cell over the gel interface, represented in Pascal.

Next, we calculated the corresponding GT displacement fields (Step 2, Fig. 1b) using Fourier Transform Traction Cytometry (FTTC), subsampling at random spatial positions within a pixel grid, where each pixel equals 30 nm, representing specific bead densities (see “Methods”). Subsequently, the fluorescent bead image pairs before and after the applied stress were generated, using the displacement values at the location of each bead and a full-width at half-maximum (FWHM) equal to that expected from the experimentally acquired lateral spatial resolution of TIRF-SIM (110 nm) or WF (270 nm) images (see “Methods”) (Step 3, Fig. 1b). The PIV analysis algorithm was then applied to the 2D TIRF-SIM and WF simulated fluorescent image pairs for a range of PIV window sizes for the single and triple square-shaped simulation stress conditions (Step 4, Fig. 1b).

The recovered displacement fields resulting from the computer simulations were then quantitatively compared to the GT displacement fields by computing the distance between the singular values of the GT displacement field and the singular values of the recovered displacement field, called the singular value decomposition (SVD) quality metric (see “Methods”; Step 5, Fig. 1b). Note, large SVD quality metric values indicate a good estimation of displacement compared to the GT. Dissecting the three outlined components contributing to the sensitivity of TFM, we explored the relationship of image spatial resolution, density of fiducial marker beads, and PIV analysis window size. Explicitly, the PIV analysis was employed for simulations at two different bead densities, 12 beads per µm^2^ (minimum bead density corresponding to the experimental condition resolvable in TIRF-SIM but not in WF to facilitate comparison between the two imaging modalities), and 82 beads per µm^2^ (maximum theoretical limit representative of one bead per 12 px PIV window size for 2D TIRF-SIM PSF) (Fig. 1c), and a range of PIV window sizes scaling from 120 to 960 nm (4–32 px) (Fig. 1a, d), exploring the influence of these parameters on the ability of PIV to resolve a given displacement field. Note that for a given bead density, the number of beads per PIV window increases with the size of that window; however, to gain a robust displacement estimation, there must be a sufficient number of beads within a given PIV window. Consequently, there is a cut-off value for each bead density wherein this number becomes too low and the PIV will fail, which in this work is considered to be one bead per PIV window (Fig. 1d).

The resulting SVD quality metric highlighted the ability of the PIV analysis approach to take advantage of high bead densities imaged via 2D TIRF-SIM-TFM over WF-TFM for triple square-shaped stress zones at both bead densities of 12 beads per µm^2^ (Supplementary Fig. 1a, b) and 82 beads per µm^2^ (Fig. 1e). By analysing the SVD quality metric heat maps presented in Fig. 1e, consistent with our expectations, it was evident that the extended spatial resolution and high fluorescent bead density as offered by TIRF-SIM improved the spatial sensitivity of TFM allowing the use of smaller PIV window sizes, with a better agreement to the GT at smaller displacements and PIV window sizes (yellow regions) than those acquired in conventional WF images (red arrow, Fig. 1e). Specifically, the simulations showed better agreement with the GT for TIRF-SIM (SVD quality metric of 1) compared to WF (SVD quality metric of 0.8). This important result was further highlighted in the plot displaying the difference in the SVD quality metrics between TIRF-SIM and WF (Fig. 1e, right). The TIRF-SIM acquisition allowed for a better estimation of small-scale displacements than WF acquisition (Fig. 1e and Supplementary Fig. 1a, b), which was in agreement with previous work^31,34^. Notably, this improvement was not present for the low bead density simulations, where TIRF-SIM and WF were comparable (Supplementary Fig. 1a, b) and in the case where the average number of beads per PIV window fell below one (dashed line, Fig. 1f and Supplementary Fig. 1c). The SVD quality metric also revealed another profound limitation inherent to PIV, whereby displacements larger than the PIV window size could not be reliably estimated (solid line, Fig. 1e and Supplementary Fig. 1a, b), as well as for displacements below the spatial resolution limit, that is the case of a bead density where less than one bead per PIV window is present (SRL red line, Fig. 1e). Additionally, the SVD quality metric revealed that for triple square-shaped stress zones, when the length-scale of the maximum displacement approached the separation distance between the respective stress fields (*d* > *L*), the ability to resolve the displacements broke down (dotted line, Fig. 1e and Supplementary Fig. 1a, b). This demonstrated a fundamental limitation of TFM, where co-linear stresses cannot be well resolved if their separation is on the same length-scale as the underlying displacements. Crucially, by analysing the maximum of the SVD quality metric, an optimal PIV window size for each simulated condition in both TIRF-SIM and WF could be inferred (Fig. 1f and Supplementary Fig. 1a, b), serving as a guide for the subsequent analysis of experimental data (see “Methods”). Note the SVD quality metrics for the single square-shaped stress field are not shown separately due to reasons of limited added information value.

Aiding the interpretation of the computer-simulation results, we developed two scoring metrics, which we will refer to in the following as the accuracy and precision diagrams (Fig. 1g–i). The accuracy diagram measures the number of recovered traction objects compared to the GT traction objects (Fig. 1b). Complementarily, the precision diagram measures the maximum recovered displacement compared to the maximum GT displacement (Fig. 1b). This quantification revealed that while both 2D TIRF-SIM and WF-TFM could robustly recover the maximum displacement values (see precision diagram, Fig. 1h), small displacements of the triple square-shaped stress zones were only recoverable for small PIV windows using 2D TIRF-SIM and not WF acquisition (see accuracy diagram, Fig. 1i). This was not the case when simulating lower bead density of 12 beads per µm^2^, where the scores of both accuracy and precision diagrams dropped down (see Supplementary Fig. 2). Note, the accuracy diagram displays the fraction of simulation repeats successfully recovering the GT number of traction objects. Complementarily, the precision diagram displays the fraction of simulations correctly recovering the maximum displacement compared to the GT (see “Methods”). The failure in the accuracy diagram for the 2D TIRF-SIM condition was due to the particular nature of the simulation with the simulated stress directed along the *x*-axis. For a narrow range of simulated stresses (450–1000 Pa), the displacement resulting from three distinct objects appeared as a uniform displacement generated by a single object (Fig. 1h, i). This was verified by simulating the same conditions but with a stress field directed along the *y*-axis (Fig. 1j), highlighting the influence of the stress magnitude and direction on the traction recovery. These computer simulations demonstrated the significance of extending fiducial marker densities, spatial resolution, and optimising PIV analysis in 2D TIRF-SIM-TFM measurements. The derived SVD quality metrics provide a guide for finding the optimal parameters for the experimental image acquisition and PIV analysis in TFM for a given spatial resolution, stress field and bead density.

### Live-cell force quantification using 2D TIRF-SIM-TFM

Having established the technical capabilities of 2D TIRF-SIM-TFM, we next investigated its performance under experimental conditions probing small mechanical forces in live-cell systems over time. Aiming to highlight the biocompatibility of the technique and to investigate small and rapid force production that would be challenging to the previous TFM methodologies, we examined three different sets of measurements comparing 2D TIRF-SIM and WF image acquisition at varying temporal acquisition rates. We focused on three different biological systems during cell–substrate adherence: the well quantified model of cervical HeLa cancer cells for validation of the technique due to large force production, the model of mast immune cell activation in RBL cells for small-scale force generation under receptor–ligand binding, and the model of cell migration in primary salmon keratocytes for rapid force generation that have been shown to be involved in wound healing^21,22^. The HeLa and RBL cell lines were transduced to both stably express Lifeact-citrine, allowing via visual inspection the correlation of force generation and the dynamics of the underlying actin architectures. The primary salmon keratocytes were labelled with CellMask Green to correlate force generation with cell membrane dynamics. Excitation of the cell’s fluorescent channel (Lifeact-citrine, 516 nm excitation, 529 nm emission; CellMask Green 535 nm excitation, 522 nm emission) and the fiducial beads (580 nm excitation, 605 nm emission) was implemented using dual-colour sequential acquisition with 488 and 542 nm excitation laser lines (see “Methods”). In all measurements, a single super-resolved TIRF-SIM image frame results from the acquisition of 9 individual raw image frames (3 angles and 3 phases) and the WF image frame was formed by averaging each of the nine raw image frames. To achieve TIRF-SIM illumination at the cell–gel interface, a well-characterised standard silicone gel with a refractive index matching the sample glass coverslip was used as the TFM substrate (see “Methods”).

**Fig. 2:**
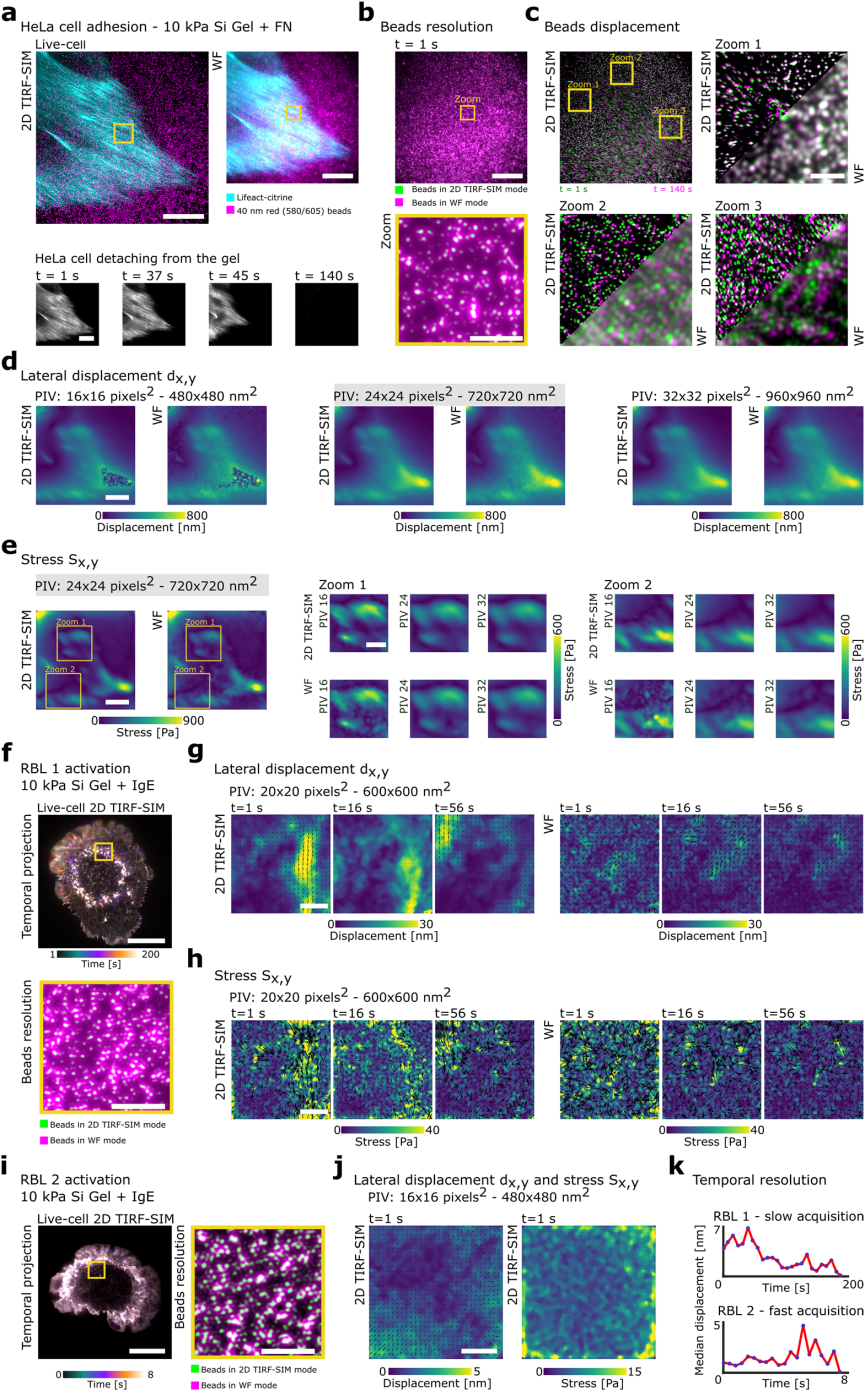
2D TIRF-SIM-TFM during detachment of adherent HeLa cells and activation of immune RBL cells. **a** Upper left: 2D TIRF-SIM imaging of a HeLa cell expressing Lifeact-citrine (cyan) on top of a 20-µm-thick silicone gel coated with fibronectin (FN) and 40 nm fluorescent beads (magenta). Upper right: Equivalent image in WF. Lower: Snap shots during its detachment. Scale bar: 10 µm. **b** Superimposed 2D TIRF-SIM (green) and WF (magenta) bead images comparing the bead resolution. Zoom-in shown below. Scale bar: 10 and 2 µm, respectively. **c** Bead positions at 1 s (green) and 140 s (magenta) superimposed highlighting the bead displacements. Three zoom-ins comparing 2D TIRF-SIM and WF imaging. Scale bar: 2 µm. **d** Lateral gel displacement generated by the HeLa cell before detaching from the gel. Optimal PIV window of 24 px. Scale bar: 10 µm. **e** Lateral stress induced by the HeLa cell before detaching, calculated with 24 px PIV window. Two zoom-ins recovered using 16, 24 and 32 px PIV windows. Scale bar: 10 and 5 µm, respectively. **f** Upper: Temporal projection of 2D TIRF-SIM imaging of RBL cell expressing Lifeact (cyan) on top of a 20-µm-thick gel coated with IgE and 40 nm fluorescent beads (magenta) acquired at 0.25 fps. Scale bar: 10 µm. Lower: Zoom-in of the bead distribution in TIRF-SIM (green) and WF (magenta). Scale bar: 5 µm. **g** Lateral displacements of the entire FOV resulting from RBL cell activation using a two frames average and a temporal resolution of 0.125 fps. PIV window used is 20 px. Vector map overlayed. Scale bar: 10 µm. **h** Its corresponding lateral stress. Vector map overlayed. Scale bar: 10 µm. **i** Left: Temporal projection of 2D TIRF-SIM imaging of RBL cell imaged acquired at 2.5 fps. Scale bar: 10 µm. Right: Zoom-in of the bead distribution in 2D TIRF-SIM (green) and WF (magenta). Scale bar: 5 µm**. j** Absence of lateral displacement and stress during 2.5 fps TIRF-SIM imaging of RBL cell activation. PIV window used is 16 px. Scale bar: 10 µm. **k** Line plots showing the overall median displacement per frame for both intermediate and rapid TIRF-SIM acquisition during RBL cell activation.

Following standard incubation protocols, the HeLa cells were allowed to adhere overnight to a 10 kPa gel substrate functionalised with fibronectin and loaded with red 40 nm fluorescent beads at a density of 12 beads per μm^2^ (Fig. 2a, b). Because the TIRF-SIM lateral resolution is 110 nm, fluorescent beads with a diameter smaller than this value were chosen to take advantage of the high-spatial resolution. Specifically, 40 nm fluorescent beads were chosen due to their small size yet bright and stable fluorescent signal, enabling both high-resolution force mapping with minimal photo-bleaching. We started to quantify mechanical force production at the ventral contact of HeLa cells during cell–gel detachment in response to treatment with 0.05% trypsin-EDTA, focusing on shear stresses *S*_*x*,*y*_ scaling in the range of 0–1 kPa. We chose to acquire time-lapses at a moderate frame rate of 0.25 image frames per seconds (fps) over a total duration of 140 s, allowing quantification throughout the complete retraction process of the HeLa cells from the gel substrate (Fig. 2a, b and Supplementary Movie 1). Aligned with previous measurements^13,38,40^, we observed the relaxation of a lateral gradient of mechanical loading across the field of view (FOV) accompanied by individual adhesion contacts relaxing as the HeLa cell detached from the surface. This is highlighted by the temporal projections of the fluorescent bead channel, and by overlaying the initial and final image frames of the TIRF-SIM and corresponding WF acquisitions (Fig. 2c). Applying the findings from the computer simulations, we analysed the experimental fluorescent displacement distributions for three PIV window sizes (Fig. 2d). Consistent with visual inspection of the simulated SVD quality metric (Supplementary Fig. 1a, b), the PIV analysis confirmed the optimal choice of 720 nm × 720 nm PIV window size for an overall maximum displacement of 800 nm (Fig. 2d and Supplementary Fig. 1c). In contrast, a selection of a smaller PIV window size, such as 480 nm × 480 nm, underestimated the magnitudes and distribution of the recovered displacement fields, as was apparent in the image region closest to the cell edge (Fig. 2d). The recovered displacement with larger 960 nm × 960 nm PIV window size was visually comparable to the one recovered with PIV window size of 720 nm. Because a larger PIV window size induces more displacement averaging, we decided to choose the 720 nm PIV window as the optimal one (Fig. 2d). Visual comparison of the cell context as provided by the Lifeact channel and the recovered displacement fields supported these findings, as did the resulting stress fields with a maximum stress of *S*_*x*,*y*_ of 900 Pa (Fig. 2e and Supplementary Movie 2). As for the recovered displacement fields, while small PIV windows allowed a more accurate recovery of smaller details within the stress field, large windows induced a smoothing effect. For all three selections of PIV window sizes, TIRF-SIM did not provide enhancement in the force magnitude, as expected due to large displacement and force generation, but still delivered improved results in terms of signal-to-noise over WF image acquisition (Fig. 2d, e and Supplementary Fig. 2a, b).

Exploring the sensitivity of 2D TIRF-SIM-TFM with smaller force regimes and higher beads densities, we next quantified mechanical force production in rapidly activating RBL cells at an experimental setting focusing on mechanical stress *S*_*x*,*y*_ in the range of 0–40 Pa. Exploiting the previously optimised protocol of living RBL cell adhesion, we allowed the RBL cells expressing the Fcε receptor-1 (FcεRI) to interact with a 10 kPa gel substrate functionalised with FcεRI-specific antibody IgE and loaded with red 40 nm fluorescent beads at a density of 15 beads per μm^2^. Employing imaging parameters comparable to the HeLa cell experiments, we consistently observed the radial spreading of the RBL cells driven by the dynamics of the cytoskeletal actin polymerisation in response to IgE-mediated activation, as displayed by temporal projections of the fluorescent cell (Fig. 2f and Supplementary Movie 3). To recover the small nm-scale displacements, we analysed the displacement between every other fluorescent bead image, reducing the temporal resolution to 0.125 fps. We observed a general contraction underneath the cell with a shear gradient of mechanical loading across the entire FOV accompanied by individual displacement features at the ventral interface of the RBL cells (Fig. 2g), as they continuously formed fresh adhesion contacts as displayed in the time-lapse images and the respective displacement fields over time. Analysis confirmed the optimal choice of 600 nm × 600 nm PIV window size for a given maximum displacement of approximately 30 nm to maintain at least one bead per PIV window (Fig. 2g and Supplementary Fig. 1c). Visual comparison of the cell context and the recovered displacement fields supported these findings, as they were also reflected in the resulting stress fields with a maximum stress of 40 Pa (Fig. 2h and Supplementary Movies 4 and 5). Notably, the TFM experiments using WF image acquisition resulted in the total loss of all spatial force features displaying only background noise in the displacement and stress fields (Fig. 2g, h and Supplementary Fig. 2a, b). Extending the temporal resolution, we quantified mechanical force production in rapidly activating RBL cells at an experimental setting focusing on comparable mechanical stress *S*_*x*,*y*_ conditions (Fig. 2i). Acquisition at a high frame rate of 2.5 fps over a total duration of 8 s resulted in background noise but no detectable displacement distribution in the TIRF-SIM and WF image acquisitions (Fig. 2j and Supplementary Movie 6), suggesting that the image frame rate exceeded the biological time-scale of cellular force production (Fig. 2k). Visual comparison of the cell context and the recovered displacement field further strengthened the notion that force was seemingly absent as no motion was visible or detectable in both the Lifeact and fiducial bead channels.

**Fig. 3:**
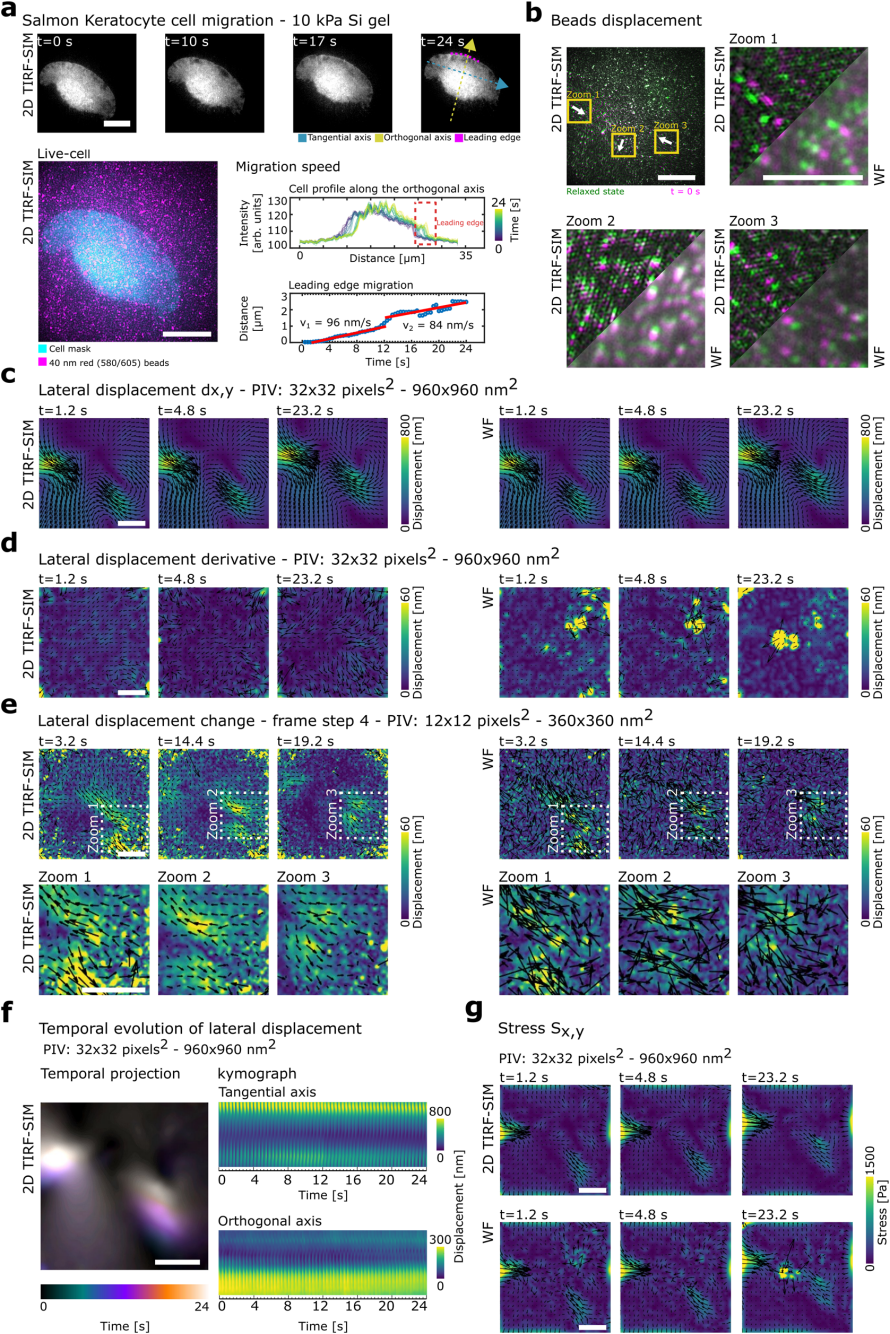
2D TIRF-SIM-TFM during migration of live salmon keratocytes. **a** Upper: Snap shots of a primary salmon keratocyte over the course of 24 s while migrating on top of silicone. Cell leading edge (magenta), the orthogonal axis (yellow) and the tangential axis (light blue) to the leading edge represented at 24 s. Lower left: 2D TIRF-SIM image of salmon keratocyte labelled with CellMask Green (cyan) on top of the gel coated with 40 nm fluorescent beads (magenta). Lower right: Cell plot profile along the orthogonal axis over migration. Dropping down in intensity of the line profiles due to the leading edge of the cell (red box). Scatter plot of the leading edge position and cell migration velocity estimated from the curve slope. Scale bar: 10 µm. **b** Bead positions at the relaxed state of the gel (green) and 0 s (magenta) superimposed to highlight the bead displacements. Scale bar: 10 µm. The zoom of three areas shows the differences between 2D TIRF-SIM and WF imaging and displacement magnitude heterogeneity. Scale bar: 5 µm. **c** Lateral displacement of the gel generated by the salmon keratocyte at time 1.2, 4.8 and 23.2 s, recovered with 2D TIRF-SIM-TFM and WF-TFM. PIV window used is 32 px. Vector map overlayed. Scale bar: 10 µm. **d** Derivative of the displacement calculated using a PIV window of 32 px (see panel **c**) showing the absence of small displacements detected, with 2D TIRF-SIM-TFM and WF-TFM. Vector map overlayed. Scale bar: 10 µm. **e** Upper: Lateral change of the displacement extracted using a PIV window of 12 px and a step of 4 frames, for both 2D TIRF-SIM-TFM and WF-TFM. Vector map overlayed. Scale bar: 10 µm. Lower: Zoom-in of the gel structure under stress. Scale bar: 10 µm. **f** Temporal evolution of the lateral displacement of salmon keratocyte during migration recovered in 2D TIRF-SIM-TFM represented by its temporal projection (left), and its kymograph along the tangential and orthogonal axes (right). Minor ticks in the time axis represent single super-resolved frames acquired in 0.4 s (2.5 fps). **g** Corresponding lateral stress recovered with 2D TIRF-SIM-TFM and WF-TFM. PIV window used is 32 px. Scale bar: 10 µm.

Lastly, to underline the combination of spatial and temporal sensitivity of the 2D TIRF-SIM-TFM, we quantified mechanical force production during primary salmon keratocyte cell migration on top of gel coated with red 40 nm fluorescent beads at a density of 15 beads per μm^2^ (Fig. 3a and Supplementary Movie 7). Salmon keratocytes present a range of different morphologies, which dictate their migration speed^22,41^. We quantified force generation of the fast-moving single keratocytes cells. To obtain single cells, salmon scales were allowed to adhere to 10 kPa gels overnight. After 24 h keratocytes in the form of a cell sheet started to migrate from the scale onto the gel, ultimately resulting in the migration of single cells which separated from the cell layer (see “Methods”). The migration of fast-moving keratocyte was acquired with a high frame rate of 2.5 fps over a total duration of 24 s (Fig. 3a). Because these are primary cells, we decided to minimise photo-toxicity by having a 5 s interval after 30 dual-colours super-resolved frames. A migration velocity of 100 nm/s was observed by plotting the cell profile along the orthogonal axis of the cell and tracking the displacement of leading edge of the cell over time, similar to previous work^22,41^ (Fig. 3a) (see “Methods”). In line with previous work, we observed forces underneath the lateral cell extremities^37^. This is underlined by overlaying the relaxed state and the first frame of the gel acquired in both 2D TIRF-SIM and WF (Fig. 3b). Zooms 1 and 3 show large bead displacements underneath the lateral cell extremities pointing towards the cell body, while zoom 2 shows smaller bead displacement at the back of the cell outwards from the cell body (Fig. 3b, white arrows). Because of the heterogeneous displacement range, the displacement and stress fields were recovered using a range of PIV windows from 12 to 32 pixels (360–960 nm) (Fig. 3c, f). The temporal evolution of the displacement and stress fields resulted in a maximum displacement of 800 nm and a stress of 1500 Pa underneath the lateral cell edge, similar to those observed in previous studies^36,37^ (Fig. 3c, g and Supplementary Movie 8). Consistent with our simulations, small PIV windows enhance spatial information compared to large PIV windows but fail in estimating large displacement values (Supplementary Fig. 3). To capture large displacements, a PIV window size of 32 px was selected to recover the overall displacement compared to the relaxed gel state (Fig. 3c and Supplementary Movie 8). To assess the temporal evolution of fluctuations that may exist in the force generation over time, we analysed the derivative of the displacement maps. Surprisingly, the resulting derivative displacement map was dominated by noise, suggesting the scale of the displacement between frames was extremely small, with the exception of frame = 30 where a 5 s interval was taken to minimise photo-toxicity, therefore registering larger values in the displacement derivate (Fig. 3d and Supplementary Movie 9). To further investigate this, we analysed the small change of the displacement by selecting from the 2.5 fps acquired data, frames separated by an interval of four frames reducing the time resolution to 0.6 fps (see “Methods”). Crucially, this analysis revealed small-scale displacements showing both contraction and relaxation of the gel substrate with a vortex-like shape underneath the lateral cell edge in the TIRF-SIM imaging (Fig. 3e left, top panel and bottom panel for the corresponding zoomed areas and Supplementary Movie 10), while the WF analysis was again dominated by noise (Fig. 3e, right, top panel and bottom panel for the corresponding zoomed areas and Supplementary Movie 10) (Fig. 3e). The temporal projection and the kymograph of the displacement field along the orthogonal and tangential axes show the fast evolution of the beads displacement underneath the cell that was possible to capture thanks to the fast 2.5 fps acquisition speed (Fig. 3f).

The three settings of HeLa, RBL and primary salmon keratocyte cell experiments demonstrated the technical capability and experimental feasibility of 2D TIRF-SIM-TFM to extend the sensitivity of force estimations to the sub-micron length-scale and sub-second time-scale, both in cell lines and primary cells. Thanks to the high spatiotemporal resolution of the 2D TIRF-SIM-TFM methodology we could observe small-scale force generation during activation of RBL cells and rapid large- and small-scale force generation during migration of primary salmon keratocyte cells, mechanical force regimes hidden to conventional TFM methodologies.

## Discussion

Quantifying mechanical force at physiological sensitivity is essential for the understanding of its influence on the function of living cells in the context of mechanobiology. In this work, we build upon recent advances in force quantification by combining TFM and 2D TIRF-SIM. The 2D TIRF-SIM-TFM methodology provides super-resolved structural information of the living cell, the cellular context of force production, as well as directionality and magnitudes of cell-generated force production. Owing to the >2-fold enhancement in spatial resolution of TIRF-SIM acquisition compared to WF, this technology facilitates the usage of smaller PIV window sizes while maintaining the fidelity of displacement, and hence stress, recovery. At the same time, the WF nature of TIRF-SIM allows for rapid acquisition at this increased spatial resolution, improving >10-fold temporally when compared to scanning based super-resolution techniques. Extending the spatial localisation power allowed the recovery of displacements of a few tens of nanometres in 2D TIRF-SIM-TFM but failed in WF-TFM. Applying TIRF-SIM illumination thus overcomes the restricted sensitivity of conventional TFM technology by extending the spatiotemporal resolution to the sub-micron length-scale and sub-second time-scale.

Computer simulations highlighted the experimental power and technical flexibility of 2D TIRF-SIM-TFM. Quantitative analysis of the simulations resulted in the SVD quality metric providing a guide for informing the design and analysis strategy of TFM experiments by finding the optimal setting for the fiducial marker bead density, experimental image acquisition parameters, and PIV analysis parameters at a given spatial resolution as provided by 2D TIRF-SIM-TFM and WF-TFM. While low bead densities give an almost comparable result between 2D TIRF-SIM and WF, high bead densities together with PIV analysis ensure maximal benefits for extended spatiotemporal resolution. In contrast to SPT analysis, individual beads are not required to be tracked in the PIV analysis, but ensembles of beads collectively inform stress recovery. Generally, smaller PIV window sizes, containing more than one bead, increase the sensitivity of force recovery. Nonetheless, this guideline breaks down when the displacements are larger than the distance between traction objects, leading to overlapping stress fields at insufficient spatial resolution. Choice of a PIV window size equal or smaller than the maximum displacement in the displacement distribution field ensures successful recovery of the cellular stress (*d* < PIV window size), which at the extreme of small sub-micron scaled displacements must be balanced by the at least one bead per PIV window.

The 2D TIRF-SIM-TFM methodology can be straightforwardly expanded to 3D force probing by combining instead of TIRF illumination the 3D SIM acquisition with axial piezo scanning^17^, but consideration should be given to the associated increase in light exposure and decrease in temporal resolution due to slow multi-frame *z*-stack acquisition. Nevertheless, in 2D TFM, TIRF illumination is superior to 2D SIM because axial fluorescence sampling restricts itself to concise isotropic sectioning as offered by the TIRF PSF in contrast to the non-isotropic 2D SIM PSF. High TIRF fluorescence illumination in 2D TIRF-SIM-TFM is thus focused on the cell–gel interface within a single image plane, minimising out-of-focus cytoplasmic fluorescence background and noise contributions.

Applying 2D TIRF-SIM-TFM to three different biological cell systems revealed the evolution of mechanical forces over time in adhering HeLa cells with respect to the relaxed state of the gel after cell detachment, in activating RBL cells and in migrating primary salmon keratocyte cells, consistent with previous studies^17,31,33,37^. The spatiotemporal sensitivity of 2D TIRF-SIM-TFM allowed the measurement of sub-micron shear displacements at the millisecond time-scale. For HeLa cells, the stress (*S*_*x*,*y*_ = 1–900 Pa, 1–900 pN/µm^2^) was recovered using 2D TIRF-SIM-TFM and WF-TFM at a comparable level using PIV analysis with a large 24 px window size. This analysis can be comparable to a SPT method together with a low bead density to avoid loss of particle tracking due to the large bead displacement. Analysis with a small PIV window revealed better recovery of structures underneath the focal adhesion of the cells. For the RBL cells, 20-fold smaller stresses (*S*_*x*,*y*_ = 1–20 Pa, 1–20 pN/µm^2^) generated during their immune activation were only visible with 2D TIRF-SIM-TFM and a PIV window size of 20 px and 12 beads/µm^2^. Finally, for the primary salmon keratocytes, while the overall large displacement compared to the relaxed state was recovered using both 2D TIRF-SIM-TFM and WF-TFM, showing a range of stresses between 1 and 1500 Pa (1–1500 pN/µm^2^), only TFM with 2D TIRF-SIM allowed to detect small gel displacements contracting and relaxing over time with a vortex-like shape, highlighting the experimental power and the biological demand of sufficient spatiotemporal sensitivity of 2D TIRF-SIM-TFM force probing.

Future advances in imaging modalities should focus on combing a reference-free methodology, equivalent to nanopatterning techniques^28,29^, that is compatible with super-resolved imaging, maximising the benefits of both approaches. Another new application to increase even further the spatial resolution of 2D TIRF-SIM-TFM would be combining this methodology with multicolours fluorescent beads where highly dense fluorescent bead images at separated fluorescence channels are acquired and then overlayed for TFM analysis with high-spatial spensitivity^10^. Additionally, further improvements could be made by introducing axial sampling at extended spatial resolution while maintaining the high temporal resolution as introduced by TIRF-SIM and increased lateral spatial resolution. This would allow both dense time information on the evolution of stress and take advantage of the additional axial sampling of stress distributions. One complementary and promising TFM methodology approaches this implementation by combining TIRF-SIM with astigmatism^35^, allowing for sensitive 3D force probing. Finally, another future advance would be extending the 2D TIRF-SIM-TFM methodology from single cell to tissue-level, combining the already established technology^42,43^.

## Methods

### Cell culture

RBL-2H3 clone cells (CRL-2256, ATCC, USA; mycoplasma tested) and HeLa cells (Product 93021013, Sigma-Aldrich; mycoplasma tested) were cultured at 37 °C in 5% CO_2_. RBL cells were cultured in minimum essential media (MEM) (Sigma-Aldrich) containing 15% fetal bovine serum (FBS), 10 mM HEPES (Lonza, UK), 2 mM l-glutamine and 1% penicillin–streptomycin. HeLa cells were cultured in Dulbecco’s modified Eagle’s medium (DMEM; Sigma-Aldrich) supplemented with 10% FBS, 2 mM l-glutamine and 1% penicillin–streptomycin. HeLa cells were transfected accordingly to the protocol^7^. Cells were split every 2 days at a volume ratio of 1:5. 24 h prior to TFM experiments, RBL cells were treated with 0.05% Trypsin-EDTA (Lonza), facilitating their detachment from the cell culture flask. Cells were then transferred to a rotating chamber at 37 °C in 5% CO_2_ to maintain their suspension state prior to experiments. Salmon keratocytes were cultivated in sterile medium consisting of Hanks' balanced salt solution (HBSS; Mediatech, Inc., A Corning Subsidiary, Manassas, VA), supplemented with 1 µg/ml amphotericin B, 100 µg/ml streptomycin, and 100 IU/ml penicillin^44^. For the fish cells, Atlantic salmon, Salmo salar L, were reared in marine water (salinity 3.5%) at 12 °C (Tromsø Aquaculture Research Station, Tromsø, Norway), and were fed with commercial salmon feed pellets (Skretting AS, Norway). Fish from 1.5 to 2 years old, each weighing 1200–1600 g, were sacrificed by an overdosage of benzoak vet (ACD Pharmaceuticals AS, Norway) prior to cell harvesting, which is allowed according to Norwegian Regulations for use of animals in experimentation (https://lovdata.no/dokument/SF/forskrift/2015-06-18-761#KAPITTEL_10). This method is also in compliance with corresponding EU legislation—Directive 2010/63/EU (https://eur-lex.europa.eu/legal-content/EN/TXT/?uri=CELEX:32010L0063). Approximately 120 mg/L water were used to kill the salmon and transported to the laboratory in a plastic tray. Individual scales were removed from the dorsal and ventral region of the fish with forceps and placed on a glass bottom Petri dish. The scales were allowed to dry for 30 s to attach to the surface of the Petri dish before adding the medium. Medium was exchanged every 48 h. For the TFM experiments, salmon scales were placed on top of the gels (insert already into the imaging holder) overnight with HBSS to let them adhere. After 24 h keratocytes in the form of a monolayer started to migrate from the scale onto the gel, ultimately resulting in the migration of single cells which separated from the cell layer. To label salmon keratocyte cells, 5 min prior to the experiment 1:1000 CellMAsk Green (ThermoFisher, Green Plasma Membrane Stain) diluted in HBSS was added to the sample chamber containing the gel and the salmon scale. After 5 min, the incubated gels were washed three times with Leibovitz’s L-15 medium (Gibco, 21083-027). The CellMask Green was stored at −20 °C to avoid degradation. L-15 medium was used for all the imaging acquisition. The fish experiment was approved by the Norwegian Animal Research Authority (NARA).

### Silicone gel substrate preparation

High refractive index silicone substrates were fabricated following previously published work^25^. The gels were fabricated combining two components (920A and 920B, Quantum Silicons, Richmond, VA) at a 1:1.1 ratio by weight, respectively. After thorough mixing and degassing, 50 µL of the gel solution was pipetted onto an 18 mm #1.5 glass coverslip pre-treated with sulfuric acid and hydrogen peroxide and spin coated at 5000 r.p.m. for 10 s to form a 20-µm-thick layer. Gels were cured at 100 °C for 2 h, after which they were stored in phosphate-buffered saline (PBS) at 4 °C. The topmost gel surface layer was then coated with 40 nm fluorescent beads. This was achieved by treating gels with a 10% (vol/vol) solution of 3-aminopropyl trimethoxysilane (Sigma-Aldrich, UK) for 5 min (min) prior to loading the fluorescent beads. This approach provides a free amine-group on the silicone surface, allowing functionalisation via 1-ethyl-3-(3-dimethylaminopropyl) carbodiimide (EDC) (Sigma-Aldrich), a bi-functional cross-linker that binds to the free amine on the gel surface, and provides a carboxyl binding group for further functionalisation. A solution of 40 nm, red fluorescent (580/605) FluoSpheres Carboxylate-Modified Microspheres (Invitrogen, UK) at a dilution of 1:2000 was combined with EDC in ddH_2_0 to form a 100 µg/mL solution. The solution was pipetted onto the top surface of the gel at a volume of 100 µL, followed by incubation for 10 min at room temperature (RT). Next, the incubated gels were washed extensively using PBS. In the case of HeLa cells, the gels were coated with a solution of 1 mg/mL fibronectin and 100 μg/mL of EDC in PBS (Invitrogen, UK) and incubated for 10 min at RT. In the case of RBL cells, the gel surfaces were first coated with a solution containing 1 mg/mL BSA, 100 μg/mL BSA-TNP and 100 μg/mL EDC and then incubated for 1 h at RT. Next the gels were washed with PBS and then coated with 10 μg/mL of anti-TNP IgE (Clone IgE-3, BD Biosciences, 5541178). As a final step, the gels were incubated for 1 h, followed a further wash in PBS.

### TIRF-SIM and WF acquisition imaging

The 2D TIRF-SIM-TFM acquisition imaging was performed on the existing TIRF-SIM platform controlled by a custom written LabView (NI) software^33^. For the excitation, two lasers were used, one with a wavelength of 488 nm (MPB Communications Inc., 2RU-VFL-P-500-488-B1R) and the second with a wavelength of 560 nm (MPB Communications Inc., 2RU-VFL-P-500-560-B1R). TIRF-SIM microscopy is a WF imaging technique in which excitation light patterns were formed by +1 and −1 diffraction orders generated by ferroelectric liquid crystal grating element (SLM). These diffracted beams were focused at the back focal aperture of the objective lens. The placement of these focused laser beams at the back focal plane was carefully controlled in order to ensure total internal reflection of the beams at the interface separating the silicone gel and solution above. A high 1.49 NA (UAPON 100XOTIRF, Olympus) oil-immersion objective was used for the TIRF modality. In order to image both the labelled cell and beads in the TIRF-SIM mode, each excitation beam was sent through an identical optical path. To achieve TIRF-SIM illumination at the interface between the gel and the cell, both excitation lights were sent with an incident NA ranging from 1.38 to 1.41. Consequently, an incident angle of sin^−1^(1.41/1.515) = 68.5° has to be used. We estimated that TIRF-SIM is maintained if cells move the gel in the axial direction within 500 nm^35^. In this case, the incident angle of the excitation light passing through a gel thick 15 µm is 68.5 ± 1.5° and the corresponding range of NA that has to be used is 1.395–1.42. The excitation beams coming from the lasers were collimated and passed through an acousto-optic tuneable filter (AOTF, AA Quanta Tech, AOTFnC-400.650-TN). After the beams were expanded, they were passed through a phase-only modulator, a polarising beam splitter, an achromatic half-wave plate (HWP, Bolder Vision Optik, BVO AHWP3), and then reflected on a ferroelectric spatial light modulator (SLM, Forth Dimension Displays, QXGA-3DM). The SLM was used to display a grating pattern with parameters corresponding to each excitation wavelength used, in order to generate the diffraction patterns and adjust critical angles for TIRF illumination at the glass gel interface. To maximise the pattern contrast, the diffracted light was kept at s-polarisation using a polarisation rotator. For each frame, we used an acquisition time between 20 and 300 ms depending on the fluorescence signal levels and the desired frame rate, leading to a super-resolution image (3 angles, 3 phases and 2 colours, 18 frames total) every 0.4–5 s. One colour super-resolved image was reconstructed from 9 raw image frames (3 angles and 3 phases) using a reconstruction method described previously^33,45^. All experiments were performed at physiological conditions using a micro-incubator (H301, Okolabs, Italy) at 37 °C and 5% CO_2_.

### Gel displacement quantification using PIV

Quantification of the gel substrate displacements in both simulated and experimental data was carried out using well-established PIV algorithms implemented using the MATLAB image processing toolbox (MATLAB R2018b, Mathworks)^46,47^. For a given pair of fluorescent bead images, before and after the application of stress, the algorithm first divides the images into sub-regions of a defined size, known as PIV windows. Next, the relative displacement between the image pair is established by locating the minimum quadratic difference (MQD) for each PIV window,

1 $$\mathop {\sum }\limits_{{{i}} = 1}^{{N}} \mathop {\sum }\limits_{{{j}} = 1}^{{N}} \left| {{{W}}_1\left( {t_0} \right) - {{W}}_2\left( {t_0 + \Delta t} \right)} \right|,$$

where *i*, *j* denote the (*i*, *j*)th pixel of the sub-region which is of size *N*, and *W*_1_ and *W*_2_ are the pixel intensities of the two PIV windows at time *t*_0_ and *t*_0_ + Δ*t*, respectively. For each PIV window, one displacement vector is assigned, producing a displacement map across the whole FOV. Each neighbouring PIV window is overlapped by 25% of its width, ensuring robust PIV displacement analysis for both simulated fluorescent image pairs and experimental data.

### Gel displacement change quantification using PIV

To quantify the change of the gel displacement during migration of primary salmon keratocytes, the videos were subsampled with a frame step of 4 to reduce noise detection while still maintaining a bead displacement of few nanometres. Next, PIV algorithm was applied for a given pair of fluorescent bead images using a PIV window size of 12 px as explained above.

### Mechanical stress quantification via PIV

To convert the displacement field into a traction field, FTTC coupled with Tikhonov regularisation is applied^10^,

2 $$\min \left\{ {\left\| {{\hat{\mathrm{G}}{\hat {\mathrm{T}}}}_{\lambda} - {\hat{\mathrm{u}}}} \right\|^2 + \lambda ^2\left\| {{\hat{\mathrm{T}}}_{\lambda}} \right\|^2} \right\}$$

where ${\hat{\mathrm{G}}}$ is the Green’s function tensor, ${\hat{\mathrm{T}}}({{x}})$ and ${\hat{\mathrm{u}}}({{x}})$ are the Fourier transforms of the traction field and the displacement field respectively, and *λ* the optimal regularisation parameter. For each cell analysed and for each PIV window value selected, the optimal *λ* value was calculated^48^. All analysis was performed using the MATLAB image processing toolbox (MATLAB R2018b, Mathworks) in combination with ImageJ2) (ref. ^49^) to display stress/displacement fields and cells on top of the gels, respectively.

### Computer simulations for 2D TIRF-SIM-TFM and WF-TFM

To quantify the dependence of the sensitivity of 2D TIRF-SIM-TFM on the spatiotemporal resolution of TIRF-SIM image acquisition, the density of the fiducial marker beads, and the PIV analysis window size, we performed computer simulations mimicking TFM force probing measurements in the presence of a well-defined mechanical stress within the dynamic range from 250 to 10 kPa and varying PIV window sizes from 120 to 960 nm. We simulated a stress field of a well-defined shape for single and triple square traction zones within a square domain of size 5 × 5 µm^2^ assuming a linear elastic gel with Young’s modulus of 10 kPa and a Poisson ratio 0.5. The stress was constant in the *x* direction (*S*_*x*_) for both the single square-shaped stress zone of fixed size 0.2 × 0.2 µm^2^ and for the three co-linear positioned triple square-shaped stress zones of fixed size 0.2 × 0.2 µm^2^ equally separated by a distance *L* = 0.2 µm. For statistical robustness, each simulation condition for a given stress field and PIV window size was simulated 100 times, every time generating a new random distribution of beads. For the quantification and interpretation of the stress field see more details below.

### Singular value decomposition analysis and best PIV selection

Quantitative SVD quality metric analysis was executed using well-established algorithms^50^. To assess levels of image distortion in the recovered displacement fields compared to the GT displacement fields, we diagonalised the respective displacement field to extract the singular values. The distance between the singular values of the GT displacement field and the recovered displacement field was calculated using the standard SVD quality metric for both 2D TIRF-SIM or WF:

3 $${\mathrm{SVD}}_{{\mathrm{quality}}\;{\mathrm{{value}}}} = \frac{{\sqrt {\left[ {\mathop {\sum }\nolimits_{{{i}} = 1}^{{n}} \left( {{{S}}_{{i}} - {\hat{\mathrm{s}}}_{{i}}} \right)^2} \right]} }}{{{{k}}^2}},$$

where *S* are the singular values of the GT, ${\hat{\mathrm{s}}}$ are the singular values for the recovered displacement field, *k* is the image size and *n* in the number of singular values. The SVD quality metric values were then averaged over the 100 simulation repeats. For visualisation, the SVD quality metrics were converted into logarithmic scale and normalised by the global maximum SVD quality value for 2D TIRF-SIM-TFM and WF-TFM (resulting in values ranging from 0 to 1). To this end, the SVD quality metric values scored one corresponding to the perfect match between values of the GT and the recovered displacement fields. The maximum SVD quality metric value informed the choice of the optimal PIV window size selection represented graphically using the software OriginPro 9.0. The error bars represent the average difference between the minimum SVD value and the two neighbouring values.

### Accuracy and precision diagrams

For the interpretation of the computer-simulation results, we introduce two scoring metrics, the accuracy and precision diagrams. The accuracy diagram measures the fraction of simulation repeats accurately recovering the number of traction objects compared to the GT traction objects. To compute the accuracy diagram, the simulated GT and recovered displacement fields for 2D TIRF-SIM-TFM and WF-TFM were binarised according to a chosen binary threshold. This binary threshold was set in the case of the single- and triple-squared traction object such that all displacement values were larger than 80% of the average among the three displacement peaks. In practice, to locate the position of the peak displacements, a peak finding algorithm was employed along the line-profile centred on the objects. After removing small objects using the built-in MATLAB R2018b function “bwareaopen”, the number of objects present in the recovered displacement field was extracted using “bwlabel” and “regionprops” for 2D TIRF-SIM and WF. This was run for each stress value and PIV window size over the 100 simulation repeats. To this end, the accuracy diagram score represents the fraction of the 100 repeats where the correct number of traction objects were recovered. Following the same computation strategy, the precision diagram score represents the fraction of the 100 repeats where the maximum recovered displacement was within 20% of the GT maximum displacement.

### Migration speed

To estimate the speed of salmon keratocytes during migration, the location in time of the leading edge was used to extract its velocity. Specifically, the cell profile along the axis orthogonal to the leading edge was plotted over time, using the Matlab function “improfile”. The front leading edge is represented by a dropped down of the intensity in each of the cell profiles. This information was used to extract the leading-edge position. By applying the first derivate to each cell profile, using the Matlab function “diff”, the location of the maximum peak in the derivative plot, between 7000 and 1000 pixels, was extracted, corresponding to the location of the cell’s leading edge. By plotting all the leading-edge location values and calculating the slope from the linear fit, the cell speed was estimated.

### Statistics

The FWHM of the TIRF-SIM PSF and WF PSF, respectively, were computed for at least 300 fluorescent beads over the course of at least three independent experiments. For the computer simulations, all in silico conditions and analysis were executed in 100 independent simulation repeats for each stress and each PIV window size, respectively. For the biological experiments, all HeLa, RBL and salmon keratocytes conditions and analysis were acquired in at least 20 individual cells over the course of at least three independent experiments.

### Reporting summary

Further information on research design is available in the Nature Research Reporting Summary linked to this article.

## Supplementary information

Supplementary Information

Description of Additional Supplementary Files

Supplementary Movie 1

Supplementary Movie 2

Supplementary Movie 3

Supplementary Movie 4

Supplementary Movie 5

Supplementary Movie 6

Supplementary Movie 7

Supplementary Movie 8

Supplementary Movie 9

Supplementary Movie 10

Reporting Summary

## Data Availability

A reporting summary for this article is available as a Supplementary Information file. The data that support the findings of this study are available from the corresponding author upon request.

## References

[1] Jansen KA (2015). A guide to mechanobiology: where biology and physics meet. Biochim. Biophys. Acta.

[2] Colin-York, H., Kumari, S., Barbieri, L., Cords, L. & Fritzsche, M. Distinct actin cytoskeleton behaviour in primary and immortalised T-cells. *J. Cell Sci.* 133, jcs232322 (2019). 10.1242/jcs.232322 PMC6898998 31413071

[3] Colin-York H (2019). Cytoskeletal actin patterns shape mast cell activation. Commun. Biol..

[4] Skamrahl M, Colin-York H, Barbieri L, Fritzsche M (2019). Simultaneous quantification of the interplay between molecular turnover and cell mechanics by AFM–FRAP. Small.

[5] Colin-York H (2019). Cytoskeletal control of antigen-dependent T cell activation. Cell Rep..

[6] Fritzsche M, Lewalle A, Duke T, Kruse K, Charras G (2013). Analysis of turnover dynamics of the submembranous actin cortex. Mol. Biol. Cell.

[7] Fritzsche M (2017). Self-organizing actin patterns shape membrane architecture but not cell mechanics. Nat. Commun..

[8] Hannezo E, Heisenberg CP (2019). Mechanochemical feedback loops in development and disease. Cell.

[9] Pullen RH, Abel SM (2019). Mechanical feedback enables catch bonds to selectively stabilize scanning microvilli at T-cell surfaces. Mol. Biol. Cell.

[10] Sabass B, Gardel ML, Waterman CM, Schwarz US (2008). High resolution traction force microscopy based on experimental and computational advances. Biophys. J..

[11] Roca-Cusachs P, Conte V, Trepat X (2017). Quantifying forces in cell biology. Nat. Cell Biol..

[12] Polacheck WJ, Chen CS (2016). Measuring cell-generated forces: a guide to the available tools. Nat. Methods.

[13] Colin-York H, Eggeling C, Fritzsche M (2017). Dissection of mechanical force in living cells by super-resolved traction force microscopy. Nat. Protoc..

[14] Korobchevskaya K, Lagerholm B, Colin-York H, Fritzsche M (2017). Exploring the potential of Airyscan microscopy for live cell imaging. Photonics.

[15] Schermelleh L (2019). Super-resolution microscopy demystified. Nat. Cell Biol..

[16] Schermelleh L, Heintzmann R, Leonhardt H (2010). A guide to super-resolution fluorescence microscopy. J. Cell Biol..

[17] Colin-York H (2019). Spatiotemporally super-resolved volumetric traction force microscopy. Nano Lett..

[18] Huse M (2017). Mechanical forces in the immune system. Nat. Rev. Immunol..

[19] Basu R (2016). Cytotoxic T cells use mechanical force to potentiate target cell killing. Cell.

[20] Hu KH, Butte MJ (2016). T cell activation requires force generation. J. Cell Biol..

[21] Sveen, L. R. et al. Wound healing in post-smolt Atlantic salmon (Salmo salar L.). *Sci. Rep*. **3565**, 1–16 (2019). 10.1038/s41598-019-39080-x PMC6400935 30837496

[22] Okimura, C., Taniguchi, A., Nonaka, S. & Iwadate, Y. Rotation of stress fibers as a single wheel in migrating fish keratocytes. *Sci. Rep*. **8**, 1–10 (2018). 10.1038/s41598-018-28875-z PMC6050267 30018412

[23] Paszek, M. J., Boettiger, D., Weaver, V. M. & Hammer, D. A. Integrin clustering is driven by mechanical resistance from the glycocalyx and the substrate. *PLoS Comput. Biol*. **5**, 10.1371/journal.pcbi.1000604 (2009). 10.1371/journal.pcbi.1000604 PMC2782178 20011123

[24] Schwarz US, Soiné JRD (2015). Traction force microscopy on soft elastic substrates: a guide to recent computational advances. Biochim. Biophys. Acta.

[25] Gutierrez E (2011). High refractive index silicone gels for simultaneous total internal reflection fluorescence and traction force microscopy of adherent cells. PLoS ONE.

[26] Kandow CE, Georges PC, Janmey PA, Beningo KA (2007). Polyacrylamide hydrogels for cell mechanics: steps toward optimization and alternative uses. Methods Cell Biol..

[27] Ferrari A (2019). Recent technological advancements in traction force microscopy. Biophys. Rev..

[28] Bergert M (2016). Confocal reference free traction force microscopy. Nat. Commun..

[29] Polio SR, Rothenberg KE, Stamenović D, Smith ML (2012). A micropatterning and image processing approach to simplify measurement of cellular traction forces. Acta Biomater..

[30] Banda OA, Sabanayagam CR, Slater JH (2019). Reference-free traction force microscopy platform fabricated via two-photon laser scanning lithography enables facile measurement of cell-generated forces. ACS Appl. Mater. Interfaces.

[31] Colin-York H (2016). Super-resolved traction force microscopy (STFM). Nano Lett..

[32] Stubb A (2020). Fluctuation-based super-resolution traction force microscopy. Nano Lett..

[33] Li, D. et al. Extended-resolution structured illumination imaging of endocytic and cytoskeletal dynamics. Science **349**, aab3500 (2015). 10.1126/science.aab3500 PMC4659358 26315442

[34] Gustafsson MGL (2005). Nonlinear structured-illumination microscopy: Wide-field fluorescence imaging with theoretically unlimited resolution. Proc. Natl Acad. Sci. USA.

[35] Li, D. et al. Astigmatic traction force microscopy (aTFM). *Nat. Commun.*10.1038/s41467-021-22376-w (2021). 10.1038/s41467-021-22376-w PMC8042066 33846322

[36] Hiroyasu S, Colburn ZT, Jones JCR (2016). A hemidesmosomal protein regulates actin dynamics and traction forces in motile keratinocytes. FASEB J..

[37] Messi Z, Bornert A, Raynaud F, Verkhovsky AB (2020). Traction forces control cell-edge dynamics and mediate distance sensitivity during cell polarization. Curr. Biol..

[38] Panagiotakopoulou M (2018). Cell cycle–dependent force transmission in cancer cells. Mol. Biol. Cell.

[39] Chen B-C (2014). Lattice light-sheet microscopy: imaging molecules to embryos at high spatiotemporal resolution. Science.

[40] Liu K (2013). Improved-throughput traction microscopy based on fluorescence micropattern for manual microscopy. PLoS ONE.

[41] Jurado C, Haserick JR, Lee J (2005). Slipping or gripping? Fluorescent speckle microscopy in fish keratocytes reveals two different mechanisms for generating a retrograde flow of actin. Mol. Biol. Cell.

[42] Pasqualini FS (2018). Traction force microscopy of engineered cardiac tissues. PLoS ONE.

[43] Aratyn-Schaus Y (2016). Coupling primary and stem cell-derived cardiomyocytes in an in vitro model of cardiac cell therapy. J. Cell Biol..

[44] Dalmo, R. A. Atlantic salmon (Salmo salar L.) epidermal malpighian cells-motile cells clearing away latex beads in vitro. *J. Mar. Biotechnol.* https://www.researchgate.net/publication/225539473 (1998).

[45] Gustafsson MGL (2008). Three-dimensional resolution doubling in wide-field fluorescence microscopy by structured illumination. Biophys. J..

[46] Raffel, M., Willert, C. E., Wereley, S. T. & Kompenhans, J. *Particle Image Velocimetry: A Practical Guide* (Springer, 1998).

[47] Keane RD, Adrian RJ (1992). Theory of cross-correlation analysis of PIV images. Appl. Sci. Res..

[48] Schwarz US (2002). Calculation of forces at focal adhesions from elastic substrate data: the effect of localized force and the need for regularization. Biophys. J..

[49] Schindelin J (2012). Fiji: an open-source platform for biological-image analysis. Nat. Methods.

[50] Shnayderman A, Gusev A, Eskicioglu AM (2006). An SVD-based grayscale image quality measure for local and global assessment. IEEE Trans. Image Process..

