# Supplementary material for: Two-dimensional TIRF-SIM–traction force microscopy (2D TIRF-SIM-TFM)
Source: Nat Commun. 2021 Apr 12;12:2169. doi: 10.1038/s41467-021-22377-9 (PMC8041833; doi:10.1038/s41467-021-22377-9)
Supplement: Supplementary file 1 — Supplementary Information [file 41467_2021_22377_MOESM1_ESM.pdf]

Supplementary Information

Two-dimensional TIRF-SIM - Traction Force Microscopy (2D TIRF-SIM-TFM)

Liliana Barbieri[1#], Huw Colin-York[1,2#], Kseniya Korobchevskaya[2#], Di Li[3], Deanna L. Wolfson[4], Narain Karedla[2,5], Falk Schneider[1,2], Balpreet S. Ahluwalia[4], Tore Seternes[6], Roy A. Dalmo[6], Michael L. Dustin[2], Dong Li[3,7], Marco Fritzsche[1,2,5*]

[1]MRC Human Immunology Unit, Weatherall Institute of Molecular Medicine, University of Oxford, Headley Way, Oxford. OX3 9DS, United Kingdom.

[2]Kennedy Institute for Rheumatology, Roosevelt Drive, University of Oxford, Oxford, OX3 7FY, United Kingdom.

[3]National Laboratory of Biomacromolecules, Institute of Biophysics, Chinese Academy of Sciences, Beijing 100101, China.

[4]Department of Physics and Technology, UiT The Arctic University of Norway, Tromsø 9037, Norway.

[5]Rosalind Franklin Institute, Harwell Campus, Didcot, OX11 0FA, United Kingdom.

[6]Norwegian College of Fishery Science, UiT The Arctic University of Norway, Tromsø, Norway.

[7]College of Life Sciences, University of Chinese Academy of Sciences, Beijing, 100049, China.

# the authors contributed equally to this work

*Correspondence to: lidong@ibp.ac.cn and marco.fritzsche@kennedy.ox.ac.uk

Supplementary Figures

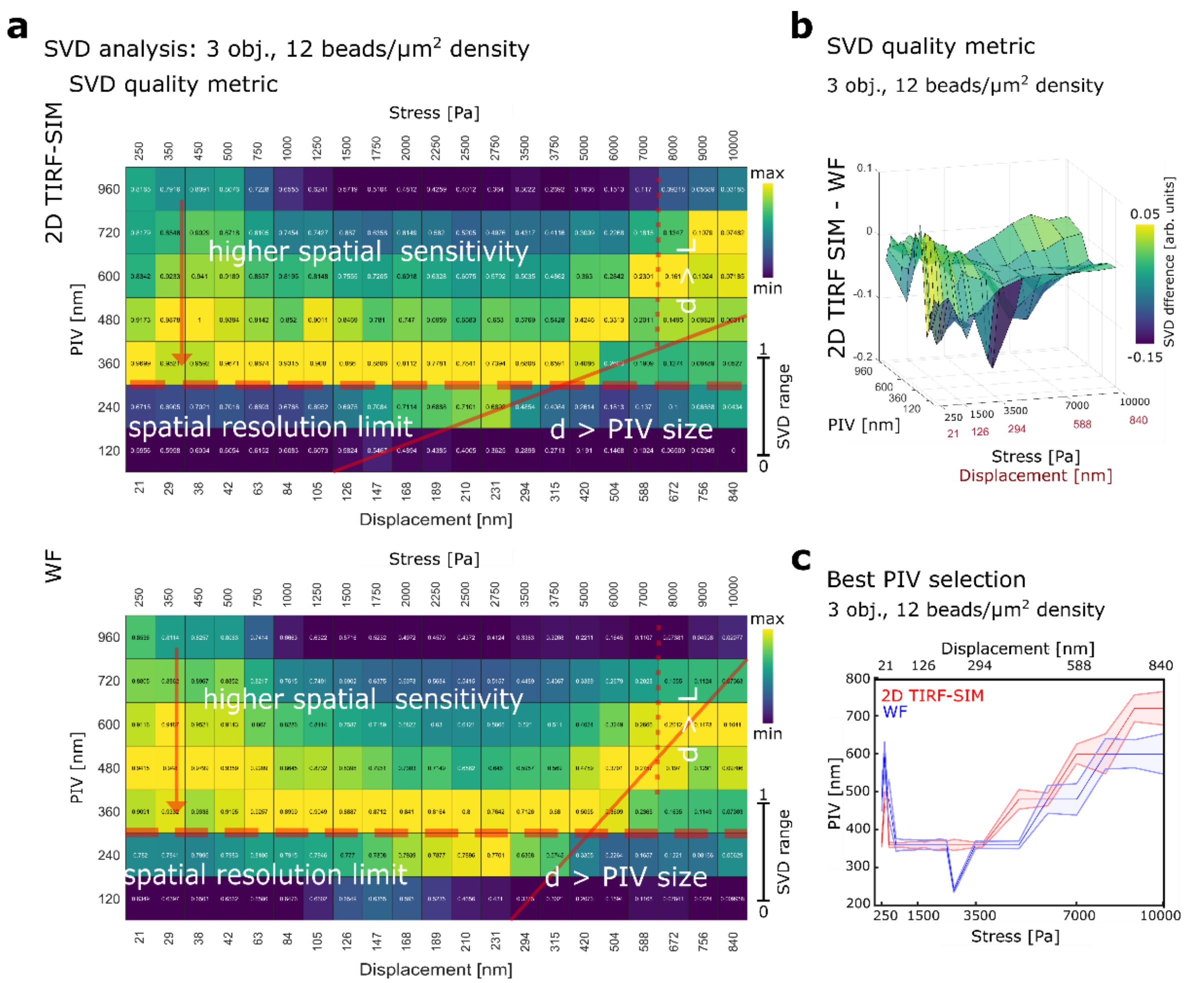

Supplementary Figure 1: SVD analysis for triple square-shaped stress zones with a bead density of 12 beads per µm². (**a**) Heatmap of the SVD analysis metric quantifying the quality of the displacement estimation compared to the GT for a range of PIV windows sizes and displacement magnitudes at bead density of 12 beads/µm² for both TIRF-SIM (left) and WF (right). The overall SVD range of values is from 0 to 1, with 1 representing the best quality of displacement estimation. Each column is colour-scaled to represent the maximum and minimum SVD values. (**b**) Difference in the SVD quality metric between TIRF-SIM and WF. All plots are normalised to the maximum SVD quality metric for TIRF-SIM, with values close to 1 indicating improved displacement estimation. (**c**) Plot highlighting the best PIV windows size for a given displacement at a bead density of 12 beads/µm², based on the minimum SVD quality metric for both TIRF-SIM (red) and WF (blue).

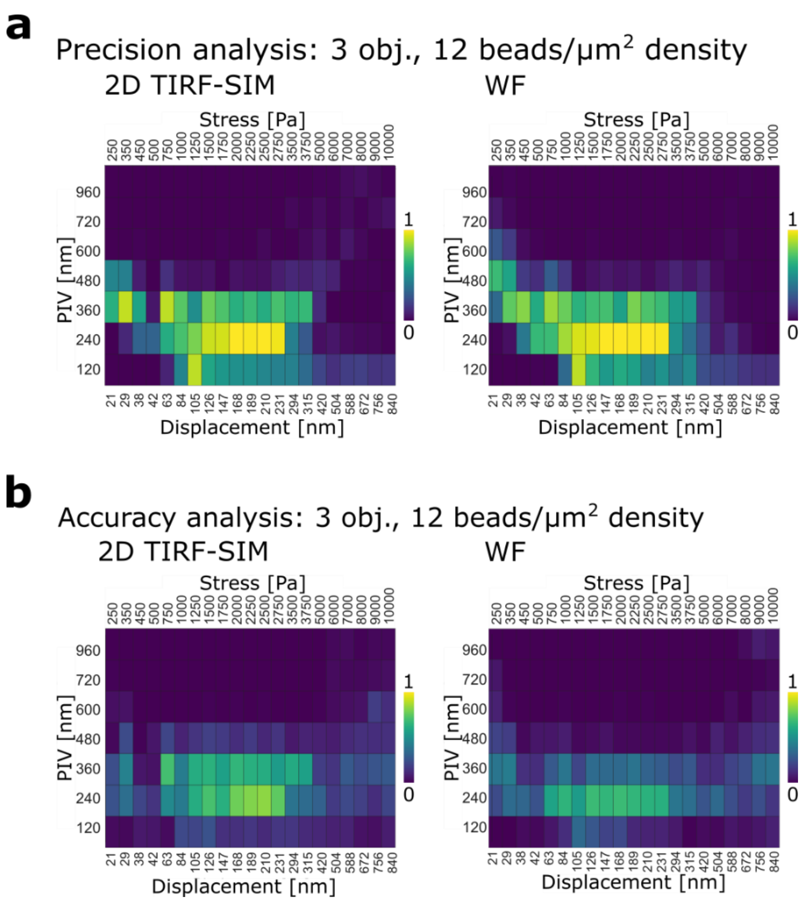

Supplementary Figure 2: Precision and accuracy diagrams for triple square-shaped stress zones with a bead density of 12 beads per µm^2. (**a**) Heatmap of the precision diagram for the case of three co-linear positioned square-shaped stress zones at a bead density of 12 beads/µm^2 for both TIRF-SIM (left) and WF (right). The values show the fraction of success among 100 repeats. (**b**) Heatmap of corresponding accuracy diagrams for the case of three co-linear positioned square-shaped stress zones at a bead density of 12 beads/µm^2 for both TIRF-SIM (left) and WF (right).

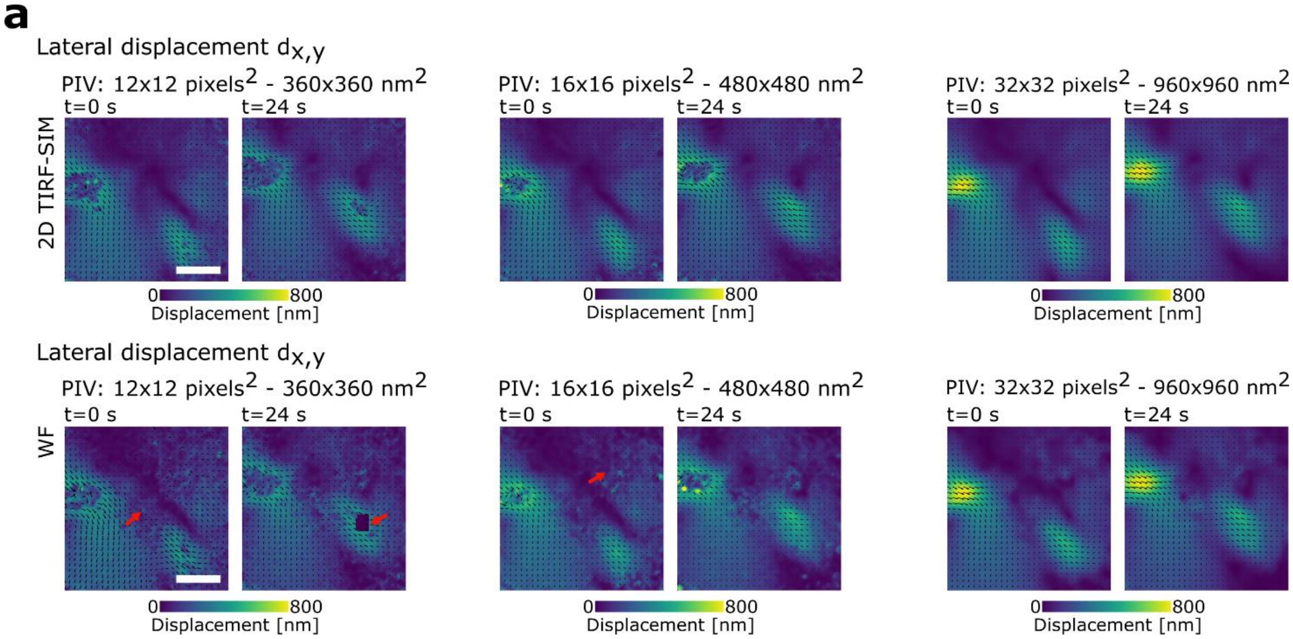

Supplementary Figure 3: Lateral displacement during migration of salmon keratocyte recovered with different PIV window size. (**a**) Lateral displacement magnitude of the gel generated by the Salmon keratocyte cell on top of the gel at time 0 and 24 sec, recovered with 2D TIRF-SIM-TFM and WF-TFM. The displacement magnitude is shown for PIV window sizes of 12, 16, and 32 px. The maximum estimated displacement was 800 nm. Optimal PIV window is chosen to be 32 px. Scale bar is 10 µm.
