# Supplementary material for: Two-dimensional TIRF-SIM–traction force microscopy (2D TIRF-SIM-TFM)
Source: Nat Commun. 2021 Apr 12;12:2169. doi: 10.1038/s41467-021-22377-9 (PMC8041833; doi:10.1038/s41467-021-22377-9)
Supplement: Supplementary file 2 — Description of Additional Supplementary Files [file 41467_2021_22377_MOESM2_ESM.pdf]

Description of Additional Supplementary Files

File Name: Supplementary Movie 1
Description: Representative time-lapse images highlighting mechanical force production indicated by red fluorescent beads (magenta) and dynamics of F-actin (cyan, Lifeact-citrine) in living HeLa cells in response to trypsin treatment acquired in TIRF-SIM (left) and WF (right) acquisition imaging. Total video duration is 140 s. The frame image rate is 0.25 fps. Scale bar is 10 µm.

File Name: Supplementary Movie 2
Description: Time series of heatmap images highlight the displacement recovery fields (top) and respective recovered stress fields (bottom) during mechanical force production in living HeLa cells in response to trypsin treatment corresponding to TIRF-SIM (left column) and WF (right column) acquisition imaging in Supplementary Video 1. The PIV window size selected is 24 px. Total video duration is 140 s. The frame image rate is 0.25 fps. Scale bar is 10 µm.

File Name: Supplementary Movie 3
Description: Representative time-lapse images highlighting mechanical force production indicated by red fluorescent beads (magenta) and dynamics of F-actin (cyan, Lifeact-citrine) during living RBL cell activation acquired in TIRF-SIM (left column) and WF (right column) acquisition imaging. Total video duration is 200 s. The frame image rate is 0.25 fps. Scale bar is 10 µm.

File Name: Supplementary Movie 4
Description: Time series of heatmap images highlight the displacement recovery fields (top) and respective recovered stress fields (bottom) during mechanical force production in activating RBL cells corresponding to TIRF-SIM (left column) and WF (right column) acquisition imaging in Supplementary Video 3. To observe the small nm-scale displacement, we looked at the displacement between every other fluorescent bead image in Video 3, reducing the temporal resolution to 0.125 fps. The PIV window size selected is 20 px. Total video duration is 200 s. Scale bar is 10 µm.

File Name: Supplementary Movie 5
Description: Time series of the stress fields overlayed with the vector field and the dynamics of F-actin during mechanical force production in activating RBL cells corresponding to TIRF-SIM (left) and WF (right) acquisition imaging in Supplementary Video 3. Stress field recovered looking at the displacement between every other fluorescent bead image in Video 3, reducing the temporal resolution to 0.125 fps. The PIV window size selected is 20 px. Total video duration is 200 s. Scale bar is 10 µm.

File Name: Supplementary Movie 6
Description: Time series of heatmap images highlight the displacement recovery fields (left) and respective recovered stress fields (right) during mechanical force production in TIRF-SIM. The PIV window size selected is 16 px. Total video duration is 8 s. The frame image rate is 2.5 fps. Scale bar is 10 µm.

File Name: Supplementary Movie 7
Description: Representative time-lapse images highlighting mechanical force production indicated by red fluorescent beads (magenta) and dynamics of cell membrane (cyan, CellMask green) during living Salmon keratocytes migration acquired in TIRF-SIM (left column) and WF (right column) acquisition imaging. Total video duration is 24 s, with a 5 sec interval after 30 dual-colours super-resolved frames acquisition, to reduce phototoxicity to the primary cells. The frame image rate is 2.5 fps. Scale bar is 10 µm.

File Name: Supplementary Movie 8
Description: Time series of heatmap images highlight the displacement recovery fields (top) and respective recovered stress fields (bottom) during mechanical force production in migrating Salmon keratocytes corresponding to TIRF-SIM (left column) and WF (right column) acquisition imaging in Supplementary Video 7. The PIV window size selected is 32 px. Total video duration is 24 s, with a 5 sec interval after 30 dual-colours super-resolved frames, to reduce phototoxicity to the primary cells. The frame image rate is 2.5 fps. Scale bar is 10 µm.

File Name: Supplementary Movie 9
Description: Time series of heatmap images highlight the derivative of the displacement recovery fields extracted using a PIV window size of 32 px (from Supplementary video 8) during mechanical force production in migrating Salmon keratocytes corresponding to TIRF-SIM (left) and WF (right) acquisition imaging in Supplementary Video 7. The frame image rate is 2.5 fps. Scale bar is 10 µm.

File Name: Supplementary Movie 10
Description: Time series of heatmap images highlight the change of the displacement recovery fields, during mechanical force production in migrating Salmon keratocytes corresponding to TIRF-SIM (left) and WF (right) acquisition imaging in Supplementary Video 7. The PIV window size selected is 12 px. The frame image rate is 0.6 fps. Scale bar is 10 µm.
