# Supplementary material for: Two-dimensional TIRF-SIM–traction force microscopy (2D TIRF-SIM-TFM)
Source: Nat Commun. 2021 Apr 12;12:2169. doi: 10.1038/s41467-021-22377-9 (PMC8041833; doi:10.1038/s41467-021-22377-9)
Supplement: Supplementary file 13 — Reporting Summary [file 41467_2021_22377_MOESM13_ESM.pdf]

nature research

Corresponding author(s): Marco Fritzsche, Dong Li

Last updated by author(s): Mar 2, 2021

Reporting Summary

Nature Research wishes to improve the reproducibility of the work that we publish. This form provides structure for consistency and transparency in reporting. For further information on Nature Research policies, see Authors & Referees and the Editorial Policy Checklist.

Statistics

For all statistical analyses, confirm that the following items are present in the figure legend, table legend, main text, or Methods section.

n/a	Confirmed	
☐	☒	The exact sample size (*n*) for each experimental group/condition, given as a discrete number and unit of measurement
☐	☒	A statement on whether measurements were taken from distinct samples or whether the same sample was measured repeatedly
☒	☐	The statistical test(s) used AND whether they are one- or two-sided<br>*Only common tests should be described solely by name; describe more complex techniques in the Methods section.*
☒	☐	A description of all covariates tested
☐	☒	A description of any assumptions or corrections, such as tests of normality and adjustment for multiple comparisons
☐	☒	A full description of the statistical parameters including central tendency (e.g. means) or other basic estimates (e.g. regression coefficient) AND variation (e.g. standard deviation) or associated estimates of uncertainty (e.g. confidence intervals)
☒	☐	For null hypothesis testing, the test statistic (e.g. *F*, *t*, *r*) with confidence intervals, effect sizes, degrees of freedom and *P* value noted<br>*Give P values as exact values whenever suitable.*
☒	☐	For Bayesian analysis, information on the choice of priors and Markov chain Monte Carlo settings
☒	☐	For hierarchical and complex designs, identification of the appropriate level for tests and full reporting of outcomes
☒	☐	Estimates of effect sizes (e.g. Cohen's *d*, Pearson's *r*), indicating how they were calculated

Our web collection on statistics for biologists contains articles on many of the points above.

Software and code

Policy information about availability of computer code

Data collection	Custom written LabView (NI) based microscope acquisition software.
Data analysis	MATLAB R2018b software and its functions regionprops, bwlabel, diff, improfile and bwareaopen; ImageJ2; OriginPro 9.0

For manuscripts utilizing custom algorithms or software that are central to the research but not yet described in published literature, software must be made available to editors/reviewers. We strongly encourage code deposition in a community repository (e.g. GitHub). See the Nature Research guidelines for submitting code & software for further information.

Data

Policy information about availability of data

All manuscripts must include a data availability statement. This statement should provide the following information, where applicable:

- Accession codes, unique identifiers, or web links for publicly available datasets
- A list of figures that have associated raw data
- A description of any restrictions on data availability

The data that support the findings of this study are available from the corresponding author upon reasonable request.

Field-specific reporting

Please select the one below that is the best fit for your research. If you are not sure, read the appropriate sections before making your selection.

☒ Life sciences ☐ Behavioural & social sciences ☐ Ecological, evolutionary & environmental sciences

For a reference copy of the document with all sections, see nature.com/documents/nr-reporting-summary-flat.pdf

October 2018

Life sciences study design

All studies must disclose on these points even when the disclosure is negative.

Sample size	The conclusions of this study do not rely on the statistical comparison of two or more groups of data, since the manuscript focuses on the description and illustration of a novel developed methodology.
Data exclusions	No data were excluded from the analysis.
Replication	Experiments were replicated independently on three different days. All samples replication were successful.
Randomization	Cells were chosen at random.
Blinding	Blinding was not undertaken in this study since the aim was to validate the performance of a new methodology looking at single-cell force production of previously investigated cell-types.

Reporting for specific materials, systems and methods

We require information from authors about some types of materials, experimental systems and methods used in many studies. Here, indicate whether each material, system or method listed is relevant to your study. If you are not sure if a list item applies to your research, read the appropriate section before selecting a response.

Materials & experimental systems

n/a	Involved in the study
☐	☒ Antibodies
☐	☒ Eukaryotic cell lines
☒	☐ Palaeontology
☐	☒ Animals and other organisms
☒	☐ Human research participants
☒	☐ Clinical data

Methods

n/a	Involved in the study
☒	☐ ChIP-seq
☒	☐ Flow cytometry
☒	☐ MRI-based neuroimaging

Antibodies

Antibodies used	10 µg/mL of Clone IgE-3, BD Biosciences, cat. n. 5541178
Validation	Validation provided by supplier

Eukaryotic cell lines

Policy information about cell lines

Cell line source(s)	HeLa Cell Line human (93021013, Sigma Aldrich), RBL-2H3 clone cells (CRL-2256, ATCC, USA), primary salmon keratocyte (Tromsø Aquaculture Research Station, Tromsø, Norway)
Authentication	The cell line and primary salmon keratocytes used in this study was not authenticated by our group.
Mycoplasma contamination	The cell line was tested and found negative for mycoplasma contamination
Commonly misidentified lines (See ICLAC register)	No commonly misidentified cell lines were used.

Animals and other organisms

Policy information about studies involving animals; ARRIVE guidelines recommended for reporting animal research

Laboratory animals	Female and male Atlantic salmon, Salmo salar L, from 1.5 to 2 years old, weighing 1200 – 1600 g.
Wild animals	*Provide details on animals observed in or captured in the field; report species, sex and age where possible. Describe how animals were caught and transported and what happened to captive animals after the study (if killed, explain why and describe method; if released, say where and when) OR state that the study did not involve wild animals.*
Field-collected samples	*For laboratory work with field-collected samples, describe all relevant parameters such as housing, maintenance, temperature, photoperiod and end-of-experiment protocol OR state that the study did not involve samples collected from the field.*

Ethics oversight | Norwegian Animal Research Authority (NARA)

Note that full information on the approval of the study protocol must also be provided in the manuscript.
